# Pole‐To‐Pole 3D Radial Trajectory Designs Improve Image Quality and Quantitative Parametric Mapping in the Brain and Heart

**DOI:** 10.1002/mrm.70237

**Published:** 2026-01-04

**Authors:** Eva S. Peper, Grzegorz Bauman, Matteo Tagliabue, Berk C. Açikgöz, Nils M. J. Plähn, Adèle L. C. Mackowiak, Yasaman Safarkhanlo, Joseph G. Woods, Davide Piccini, Li Feng, Christopher W. Roy, Oliver Bieri, Jessica A. M. Bastiaansen

**Affiliations:** ^1^ Department of Diagnostic, Interventional and Pediatric Radiology (DIPR) Inselspital, Bern University Hospital, University of Bern Bern Switzerland; ^2^ Translational Imaging Center (TIC), Swiss Institute for Translational and Entrepreneurial Medicine Bern Switzerland; ^3^ Division of Radiological Physics, Department of Radiology University Hospital Basel Basel Switzerland; ^4^ Department of Biomedical Engineering University of Basel Allschwil Switzerland; ^5^ Graduate School for Cellular and Biomedical Sciences University of Bern Bern Switzerland; ^6^ Department of Radiology Lausanne University Hospital and University of Lausanne Lausanne Switzerland; ^7^ Department of Cardiology Inselspital, Bern University Hospital Bern Switzerland; ^8^ Scientific Collaborations and Strategic Partnerships, Siemens Healthcare Srl Milano Italy; ^9^ Center for Advanced Imaging Innovation and Research (CAI2R), Department of Radiology New York University Grossman School of Medicine, New York University New York USA

## Abstract

**Purpose:**

To design 3D radial spiral phyllotaxis trajectories aimed at removing phase inconsistencies, improving image quality, and enhancing parametric mapping accuracy by acquiring nearly opposing spokes starting from both hemispheres in 3D radial *k*‐space.

**Methods:**

Two 3D radial trajectories, pole‐to‐pole and continuous spiral phyllotaxis, were developed and implemented on a 3T MRI scanner in a phase‐cycled balanced steady‐state free precession (bSSFP) and a spoiled gradient‐echo (GRE) sequence. Image quality and *k*‐space center phase variations were evaluated in a spherical phantom using the original and new radial phyllotaxis designs. T1/T2 was quantified and compared using phase‐cycled bSSFP data acquired with the new radial trajectory designs, as well as the original phyllotaxis trajectory and a Cartesian trajectory as references, in both an MRI system phantom and the brains of three healthy volunteers. ECG‐triggered whole‐heart GRE data were acquired using the original and pole‐to‐pole phyllotaxis trajectories in three healthy volunteers and compared for image quality improvement.

**Results:**

All 3D radial trajectory designs showed variations in the *k*‐space center phase depending on the orientation of the readout spokes. Image quality improved when using the pole‐to‐pole and continuous phyllotaxis over the original trajectory. Scans using the original trajectory had higher T1/T2 estimation errors in comparison to the new trajectories and the Cartesian trajectory. The pole‐to‐pole and continuous trajectories improved T1/T2 maps of the brain and image quality for all cardiac images.

**Conclusion:**

Acquiring nearly opposing spokes in 3D radial trajectory designs compensates phase inconsistencies without requiring additional corrections, which improves quantitative imaging and anatomical visualizations.

## 1 Introduction

Non‐Cartesian *k*‐space trajectories, such as 3D radial spiral phyllotaxis [1, 2], offer inherent robustness to motion and flexibility in data sorting, for example into motion bins [3], as well as *k*‐space undersampling. These properties make them well‐suited for advanced image reconstruction techniques such as compressed sensing [4]. Their value has been demonstrated for dynamic and anatomical imaging in the heart [1, 2, 5, 6], lung [7, 8], liver [9], and even the moving eye [10].

However, previous studies using 3D radial trajectories have reported signal smearing artifacts, particularly in areas with incomplete fat suppression [6, 11]. This effect has been observed across various sequences using 3D radial trajectories, including spoiled gradient‐echo (GRE) [6, 12], balanced steady‐state free precession (bSSFP) [13], and fast interrupted steady‐state (FISS) [11]. Unlike Cartesian imaging, which uses unidirectional readouts, 3D radial scans acquire spokes from different directions in *k*‐space, which makes them more prone to gradient‐induced dephasing and mismatches in *k*‐space center positions [14] caused by gradient system imperfections and eddy currents [15, 16]. This may lead to artifacts and consequently to inaccuracies in both the magnitude and phase of the reconstructed images [15].

Various 3D radial ordering schemes—such as the Archimedean spiral [17, 18], spiral phyllotaxis [2], and other trajectories [1, 19], including (double) golden angle designs [20, 21, 22]—have been proposed to ensure efficient *k*‐space coverage and smooth transitions between spokes, thereby reducing eddy current effects during acquisition. Additionally, correcting radial trajectories typically involves compensating for timing errors along the readout direction. Therefore, gradient systems can be characterized using a gradient impulse response function (GIRF) [23, 24, 25] or field cameras [26], and timing delays can be estimated via calibration scans [27] or post‐processing techniques [15, 28, 29, 30]. Other corrections can also be applied prior to the scan by adapting the sequence waveform or using pre‐emphasis modifications [24]. Although these techniques improve image quality in 3D radial acquisitions, they are not universally available, require specialized expertise, and often do not support real‐time image reconstruction on the scanner.

Another approach to reduce artifacts due to phase inconsistencies is to acquire spokes from all directions, that is not just those starting from one semicircle (2D) or hemisphere (3D), which has been demonstrated in 2D radial imaging [14, 27, 28]. The inherent cancelation of phase errors when near‐opposing spokes are combined [31, 32, 33] suggests that such a modification could be used for 3D radial imaging, for example within a full‐spoke 3D radial spiral phyllotaxis design [2], in which each spoke starts from one hemisphere in *k*‐space [2, 34]. This led to the hypothesis that a redesigned 3D radial spiral phyllotaxis trajectory that (1) acquires spokes starting from both hemispheres throughout the scan to self‐compensate for accumulated phase errors (*pole‐to‐pole phyllotaxis*), and (2) avoids jumps at the beginning of each interleave to reduce eddy current‐induced effects through continuous acquisition (*continuous phyllotaxis*), could improve both image quality and the accuracy of parametric maps without the need for additional corrections.

Apart from image artifacts, timing errors and phase variations affect the signal decoding process, such as in parametric mapping with phase‐cycled bSSFP, which has shown promise for proton density fat fraction quantification [31], quantitative T1 and T2 mapping [32, 33, 35, 36, 37, 38] and quantitative susceptibility mapping [39]. These mapping applications use complex bSSFP profiles and are therefore particularly sensitive to eddy current‐induced phase inconsistencies [40, 41].

The aim of this study was therefore to implement and evaluate the two new 3D radial trajectory designs within a phase‐cycled bSSFP and a GRE sequence to improve quantitative mapping and anatomical imaging without any further correction steps. The performance of the new trajectory designs, pole‐to‐pole and continuous 3D radial spiral phyllotaxis, was evaluated in terms of image quality and T1 and T2 mapping accuracy compared to the original 3D radial spiral phyllotaxis trajectory [2] and a conventional Cartesian trajectory. Phase‐cycled bSSFP data were acquired in a standardized MRI system phantom with known T1 and T2 reference values and in the brains of three healthy volunteers. Whole‐heart free‐breathing GRE [1, 6, 11, 42] data were acquired in the hearts of three healthy volunteers to assess the visualization of the coronary arteries.

## 2 Methods

### 2.1 3D Radial Spiral Phyllotaxis Trajectory and Definitions

**FIGURE 1:**
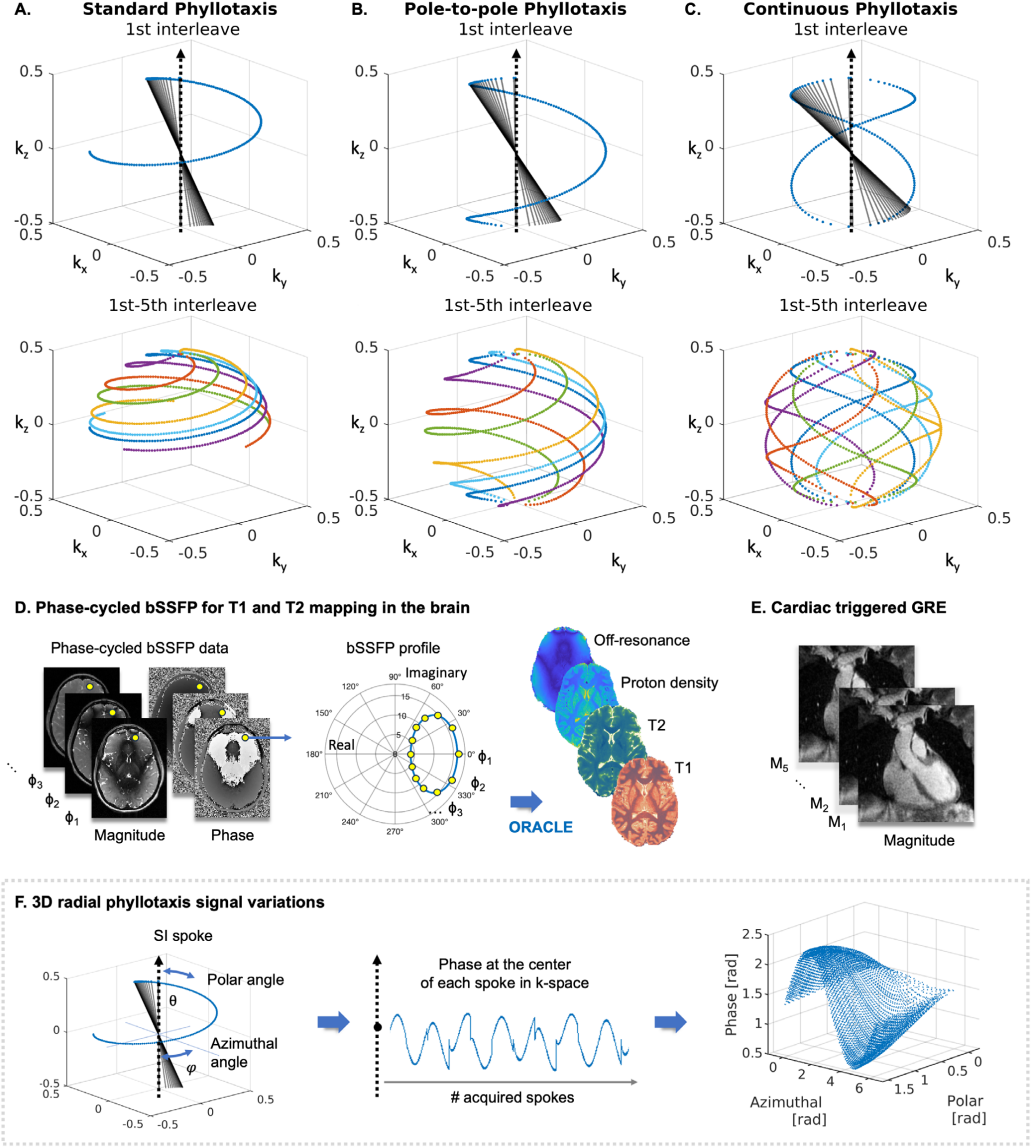
Design of the study. Normalized *k*‐space trajectories for the three variants of the 3D radial phyllotaxis trajectory: (A) original phyllotaxis, (B) pole‐to‐pole phyllotaxis, and (C) continuous phyllotaxis. The first row shows the first interleave of each acquisition, with readout starting points in blue and the full readout spokes for the first 1–20 spokes of each interleave in black. The second row visualizes the first five interleaves, highlighting the readout starting points with different colors to illustrate *k*‐space coverage. Interleaves are rotated by a golden angle increment. (D) The trajectories were used for T1 and T2 mapping in the brain using phase‐cycled bSSFP acquisitions. (E) In addition, trajectories were tested in ECG‐triggered GRE imaging of the heart. (F) Definition of polar and azimuthal angles parameterizing the 3D radial trajectory, along with signal variation extraction at the center of each spoke.

The 3D radial spiral phyllotaxis [2] pattern consists of different interleaves (Figure 1A), that are rotated by the golden angle until all readout spokes N are acquired. The positions of the n=1,…,N readout spokes of the original 3D radial spiral phyllotaxis [2] trajectory is defined in spherical coordinates by a constant radius r, an azimuthal angle φ (within the xy‐plane), and a polar angle θ (relative to the z‐axis). The azimuthal angle increases with each spoke by the golden angle $\varphi_{\mathrm{GA}}$. The azimuthal angle of the nth spoke, $\varphi_{n}$, defined within the interval [0,2π), is given by:

(1) $$\varphi_{n}=n\cdot \varphi_{\mathrm{GA}}$$

with $\varphi_{\mathrm{GA}}\approx 2.399\;\mathrm{rad}$ or 137.45 degrees. The corresponding polar angle $\theta_{n}$, defined within [0,π/2), increases with each readout according to the total number of spokes N:

(2) $$\theta_{n}=\frac{\pi }{2}\sqrt{\;\frac{n}{N}}$$

The acquisition order of the N readout spokes (Figure 1A–C, readout spokes (black) with respective starting points (blue)) is organized into K interleaves (Figure 1A–C, different colors per interleave), each containing J=N/K spokes per interleave. Each spoke n is distributed using the index of the *j*th spoke (j=1,…,J) in the kth interleave (k=1,…,K) as follows:

(3) n(j,k)=k+(j−1)·K

If *K* is a Fibonacci number, the spokes within each interleave share similar azimuthal angles, which leads to uniform *k*‐space coverage and ensures a smooth *k*‐space trajectory—meaning that the spacing between consecutively acquired spokes is minimized.

Following this approach, the starting points of all readout spokes are in the upper hemisphere. After the last spoke of an interleave is acquired, there is a jump in *k*‐space from the equator to the pole [2]. The first spoke may be used for respiratory motion extraction when aligned with the patient's superior–inferior (SI) axis. Although this spoke is conventionally referred to as the “SI‐spoke,” this designation may no longer be anatomically accurate if the field of view (FOV) is rotated. Nevertheless, the term is retained for consistency with prior literature.

### 2.2 Pole‐To‐Pole and Continuous 3D Radial Phyllotaxis Trajectory Designs

Two 3D radial trajectories were designed based on the original implementation [2]. First, the original 3D radial phyllotaxis [2] trajectory (Figure 1A, *original 3D radial phyllotaxis*) was tested, in which the polar angle θ spans the range [0,π/2), increasing from the pole to the equator of the sphere, as previously described.

To obtain a *pole‐to‐pole 3D radial phyllotaxis* trajectory, the original trajectory (section 2.1) was modified such that θ spanned the full polar range from one pole to the other, that is [0,π). This is similar to half‐radial sampling schemes [43], in which half‐spokes are acquired in all directions. The proposed (full‐spoke) trajectory (Figure 1B) is defined by:

(4) $$\theta_{n}=\left\{\begin{array}{l} \frac{\pi }{2}\sqrt{\;\frac{2n}{N}}\;\text{for}\;1\leq n<N/2\\ \pi -\frac{\pi }{2}\sqrt{\;\frac{2\left(N-n\right)}{N}}\;\text{for}\;N/2\leq n\leq N \end{array}\right.$$

In this case the polar angle θ is continuously increased, such that the readout spokes start from both the upper and lower hemisphere. A second variant, the *continuous 3D radial phyllotaxis* trajectory (Figure 1C), was designed to minimize large jumps in *k*‐space by ensuring that the trajectory returns to its starting point at the end of each interleave. In general, minimizing jumps implies that the starting points of adjacent trajectories are closer together in *k*‐space.

Here, the polar angle θ spans the full circle [0,2π), minimizing eddy current effects at the start of each interleave. The polar angles $\theta_{n}$ are therefore defined as:

(5) $$\theta_{n}=\left\{\begin{array}{l} \frac{\pi }{2}\sqrt{\;\frac{4n}{N}}\;\text{for}\;1\leq n<N/4\\ \pi -\frac{\pi }{2}\sqrt{\;\frac{4\;\left(N/2-n\right)}{N}}\;\text{for}\;N/4\leq n<N/2\\ \pi +\frac{\pi }{2}\sqrt{\;\frac{4\;\left(n-N/2\right)}{N}}\;\text{for}\;N/2\leq n<3N/4\\ 2\pi -\frac{\pi }{2}\sqrt{\;\frac{4\;\left(N-n\right)}{N}}\;\text{for}\;3N/4\leq n\leq N \end{array}\right.$$

### 2.3 Data Acquisition

A 3D radial and a 3D Cartesian phase‐cycled bSSFP sequence (Figure 1D) were implemented on a 3 T MRI scanner (MAGNETOM Prisma, Siemens Healthineers, Forchheim, Germany). Additionally, a 3D radial ECG‐triggered GRE sequence [6, 12] was used to acquire data using the original and the pole‐to‐pole trajectory (Figure 1E). For all scans, subjects gave informed consent, and the study adhered to local ethics guidelines.

In total, five experiments were conducted in phantoms and in the brain and hearts of healthy volunteers to compare the performance of the original and the new phyllotaxis trajectories. The 3D radial trajectories of all bSSFP experiments were fully sampled and included 200 spokes per interleave and 89 interleaves, resulting in a total of 17 800 spokes. All bSSFP acquisitions used 1000 dummy RF pulses to establish a magnetization steady‐state. Data was acquired at the scanner isocenter using non‐selective excitation. An overview of all five experiments and their specific acquisition parameters can be found in Table 1.

**TABLE 1:** MRI experiments and acquisition parameters.

Experiment	#1 Different scanners, FOV orientations, BWs		#2 Salt‐water phantom		#3 T1, T2 phantom		#4 In vivo brain		#5 In vivo heart	
Sequence	bSSFP		bSSFP		Phase‐cycled bSSFP		Phase‐cycled bSSFP		GRE	
TE/TR [ms]	1.8/3.6		1.8/3.6		2.5/5.0		2.5/5.0		2.6/5.5	
Matrix size	80 × 80 × 80		80 × 80 × 80		Radial: 96 × 96 × 96 Cartesian: 96 × 96 × 80		Radial: 96 × 96 × 96 Cartesian: 192 × 168 × 120		176 × 176 × 176	
Resolution [mm^3^]	2 × 2 × 2		2 × 2 × 2		1.1 × 1.1 × 1.1		1.25 × 1.25 × 1.25		1.14 × 1.14 × 1.14	
FOV [mm^3^]	160 × 160 × 160		160 × 160 × 160		Radial: 106 × 106 × 106 Cartesian: 106 × 106 × 88		Radial: 120 × 120 × 120 Cartesian: 240 × 210 × 150		200 × 200 × 200	
BW [Hz/pixel]	340 and 1389		1389		491		965		888	
RF excitation angle [°]	20		20		20		20		10	
RF phase increment [°]	180		180		0–340 in steps of 20		0–330 in steps of 30		—	
Phase cycles	1		1		18		12		—	
#interleaves, #spokes per interleave, #total number of spokes	89, 200, 17 800		89, 200, 17 800		Radial: 89, 200, 320 400		Radial: 89, 200, 213 600		547, 18, 9846	
Trajectories and overall scan time	Original phyllotaxis	1 min	Original phyllotaxis	1 min	Original phyllotaxis	25 min	Original phyllotaxis	17 min	Original phyllotaxis	∼8 min
			Pole‐to‐pole phyllotaxis	1 min	Pole‐to‐pole phyllotaxis	25 min	Pole‐to‐pole phyllotaxis	17 min	Pole‐to‐pole phyllotaxis	∼8 min
			Continuous phyllotaxis	1 min	Continuous phyllotaxis	25 min	Continuous phyllotaxis	17 min		
					Cartesian	14 min	Cartesian	9 min		

First, to showcase the signal variations across scanners, FOV orientations, and readout bandwidths for scans acquired with the original phyllotaxis trajectory, bSSFP data with a single RF phase increment of 180° was acquired in a spherical salt‐water phantom (Siemens Healthineers, Forchheim, Germany) using a 32‐channel chest and spine coil. Scans were repeated with transverse, sagittal, and coronal FOV orientations and two bandwidths (BW = 340 Hz/pixel and 1389 Hz/pixel). Additional scans were performed on three different 3 T MRI systems (MAGNETOM Prisma, Siemens Healthineers, Forchheim, Germany) using software versions VE11C and VA60. The experiment was repeated on MRI system 1 in the brain of a healthy volunteer using the same settings, to compare signal behavior of the reconstructed images between phantom and volunteer data.

In a second experiment, bSSFP data was acquired similar to the first experiment in a salt‐water phantom to compare signal behavior and artifacts in acquisitions using the three different 3D radial trajectories (Figure 1A–C). Experiments were performed with a transverse orientation and BW = 1389 Hz/pixel on MRI system 1. To investigate if the observed phase discrepancies could be corrected for using a GIRF, a GIRF [44] was acquired for MRI system 1 and first order corrections were applied to the data acquired with the original phyllotaxis trajectory. Additionally, to investigate the effect of eddy currents, the experiment was repeated using the pole‐to‐pole trajectory with each spoke acquired four times before proceeding to the next.

A third experiment was performed in a standardized 136 mini system phantom (Caliber MRI, Boulder, CO, USA) to compare T1 and T2 values quantified using a phase‐cycled bSSFP sequence with the phantom reference values. Phase‐cycled bSSFP scans were performed using the three 3D radial trajectories (Figure 1A–C) and a Cartesian trajectory. RF phase increments were increasing from 0° to 340° in steps of 20°, resulting in 18 RF phase increments. For each RF phase increment the same trajectory was repeated. The scan duration of each fully sampled phase‐cycled bSSFP scan was 25 min. The Cartesian acquisition matched these parameters.

The fourth set of MRI experiments were performed in the brains of three healthy volunteers to compare between T1 and T2 values quantified with phase‐cycled bSSFP sequences using the three 3D radial trajectories (Figure 1A–C) and a Cartesian trajectory [37] as reference. Phase‐cycled bSSFP data was acquired using a 20‐channel head coil. RF phase increments were increasing from 0° to 330° in steps of 30°, resulting in 12 RF phase increments to limit total scan time to 1 h (Figure 1D). Cartesian acquisitions were performed in sagittal orientation to avoid signal fold‐over from the abdomen.

Finally, whole‐heart MRI experiments were performed at 3 T to assess the cardiac image quality using the original (Figure 1A) and pole‐to‐pole phyllotaxis (Figure 1B) trajectories, each within an 8 min acquisition time. Data was acquired in three healthy volunteers using a free‐breathing ECG‐triggered 3D GRE sequence [6, 12] with a 2.2 ms LIBRE water excitation RF pulse for fat signal suppression [45]. The data acquisition protocol for this experiment used 547 interleaves, fitting each interleave acquisition, consisting of 18 spokes, into a diastolic window of 100 ms within each heartbeat. A T2 preparation module [46] to improve blood‐myocardium was applied prior to each interleave.

### 2.4 Image Reconstruction

All raw data were exported and processed offline in MATLAB (R2022a, MathWorks, Natick, MA, USA). The 3D radial data acquired were reconstructed using density compensation weights and a gpuNUFFT‐based [47, 48] gridding and Fourier transform. The SI‐spoke was not included during image reconstruction. Coil combination was performed on gridded data using sensitivity maps estimated via adaptive coil combination [49] from a 4 × 4 × 4 voxel region at the *k*‐space center. Similarly, the Cartesian data were reconstructed using a 3D fast Fourier transform and the same sensitivity map estimation method.

#### 2.4.1 Evaluation of Trajectory‐Dependent Signal Variations

To determine trajectory‐dependent signal variations of the second experiment, the phase ϕ at the center of the readout of the signal S defined as

(6) $$\phi^{\text{center}}=∡S_{\theta ,\varphi }$$

was plotted for each spoke and each coil against polar and azimuthal angles for the radial bSSFP data for all three radial trajectories (Figure 1F). These variations were plotted for both angles in spherical coordinates to enable spatial visualization of phase offsets relative to the physical scanner axes. Images were then reconstructed for all three trajectories as described above. Difference maps between the data acquired with each radial trajectory were calculated by subtracting the magnitude and phase images reconstructed from the original and pole‐to‐pole phyllotaxis trajectories, and the original and continuous phyllotaxis trajectories, respectively. In the acquisitions with four repeated spokes, individual reconstructions were performed for each repetition.

#### 2.4.2 Correction Method to Demonstrate Phase‐Compensation

A correction method was implemented to demonstrate the phase‐compensating effect of opposing spokes using the pole‐to‐pole trajectory. This method was used only on data of the second experiment and after sorting the pole‐to‐pole trajectory into spokes starting from the top and the bottom in the following way: (1) The pole‐to‐pole dataset was retrospectively divided into spokes starting from the upper and lower hemispheres. (2) For each spoke starting in the top hemisphere, the spatially closest opposing spoke was identified that started in the bottom hemisphere. (3) The center phase value of the opposing spoke $\phi_{\text{opposing}}$,

(7) $$\phi_{\text{opposing}}^{\text{center}}=∡S_{\theta_{\text{opposing}},\varphi_{\text{opposing}},\text{center}}$$

was added as a constant phase term to each spoke and for each coil in *k*‐space, according to

(8) $$S_{\theta ,\varphi }^{\prime }=S_{\theta ,\varphi }\;e^{-i\;\phi_{\text{opposing}}^{\text{center}}}$$

After the phase term was added, images of the phantom were reconstructed as for the other datasets as described above. A comparison of image quality of images reconstructed with and without applying the phase term was done.

#### 2.4.3 Gradient Delay Correction

A gradient delay correction method, similar to Block et al. [27], was implemented for the pole‐to‐pole phyllotaxis trajectory and applied to the data from the second experiment to demonstrate that the trajectory self‐corrects these delays. The method was implemented as follows: (1) for each spoke, the closest opposing spoke was identified; (2) the phase difference in image space per coil between each spoke and its opposing counterpart was computed; (3) the coil information was combined by summing the phase differences; (4) a linear fit in regions with sufficient magnitude signal was performed; and (5) the resulting slope a and intercept b from this fit were applied as a correction to each spoke in image space (FT denotes a 1D Fourier transform along the readout):

(9) $$S_{\theta ,\varphi }^{\prime }=\text{FT}\left(i\text{FT}\left(S_{\theta ,\varphi }\right)\;e^{-i\;\left(a*\phi +b\right)}\right)$$

#### 2.4.4 Calculation of Parametric T1 and T2 Maps

For each voxel the phase‐cycled bSSFP profile, that is the complex signal as function of the RF phase increment, exhibits tissue‐specific dependencies. The bSSFP profile appears as an elliptical [50] pattern when plotted in the complex plane. Quantitative T1 and T2 maps were estimated for each voxel using ORACLE [37], which is an analytical model that derives T1, T2, off‐resonance, and proton density from the bSSFP profile. In reconstructed images of the standardized 136 mini system phantom, regions of interest (ROIs) were drawn in each T1 and T2 compartment. The mean signal per ROI and the signal average of two adjacent slices was computed, and bSSFP signals were plotted in the complex plane across all RF phase increments, forming characteristic elliptical [50] curves. To assess the accuracy of all trajectories for T1 and T2 mapping, the estimated T1 and T2 values were plotted against the reference values, and a linear regression was performed on the log10‐transformed data. The quality of the fit was quantified by the coefficient of determination (*R* ^2^). In addition, the root mean square error (RMSE) between the estimated and reference values was calculated to assess the absolute deviation across T1 and T2 values.

#### 2.4.5 Cardiac Image Reconstruction

From the free‐breathing ECG‐triggered cardiac GRE data respiratory motion was extracted using a self‐gating algorithm [51], which derives respiratory signal variations from the SI‐spoke. Based on the self‐gating signal, the data was sorted into five respiratory motion states (Figure 1E), resulting in 5‐times undersampled datasets per motion state. Using the same gpuNUFFT [47, 48] and sensitivity map estimation functions for coil combination [49] as described above, a motion‐resolved compressed sensing image reconstruction [48, 51] was applied using a subspace model [48] and a spatial total variation regularization λ = 10^−9^ and 25 iterations. The end expiration‐state was selected and the image was reformatted using Soap‐Bubble [52] to visualize the coronary arteries for all subjects.

## 3 Results

**FIGURE 2:**
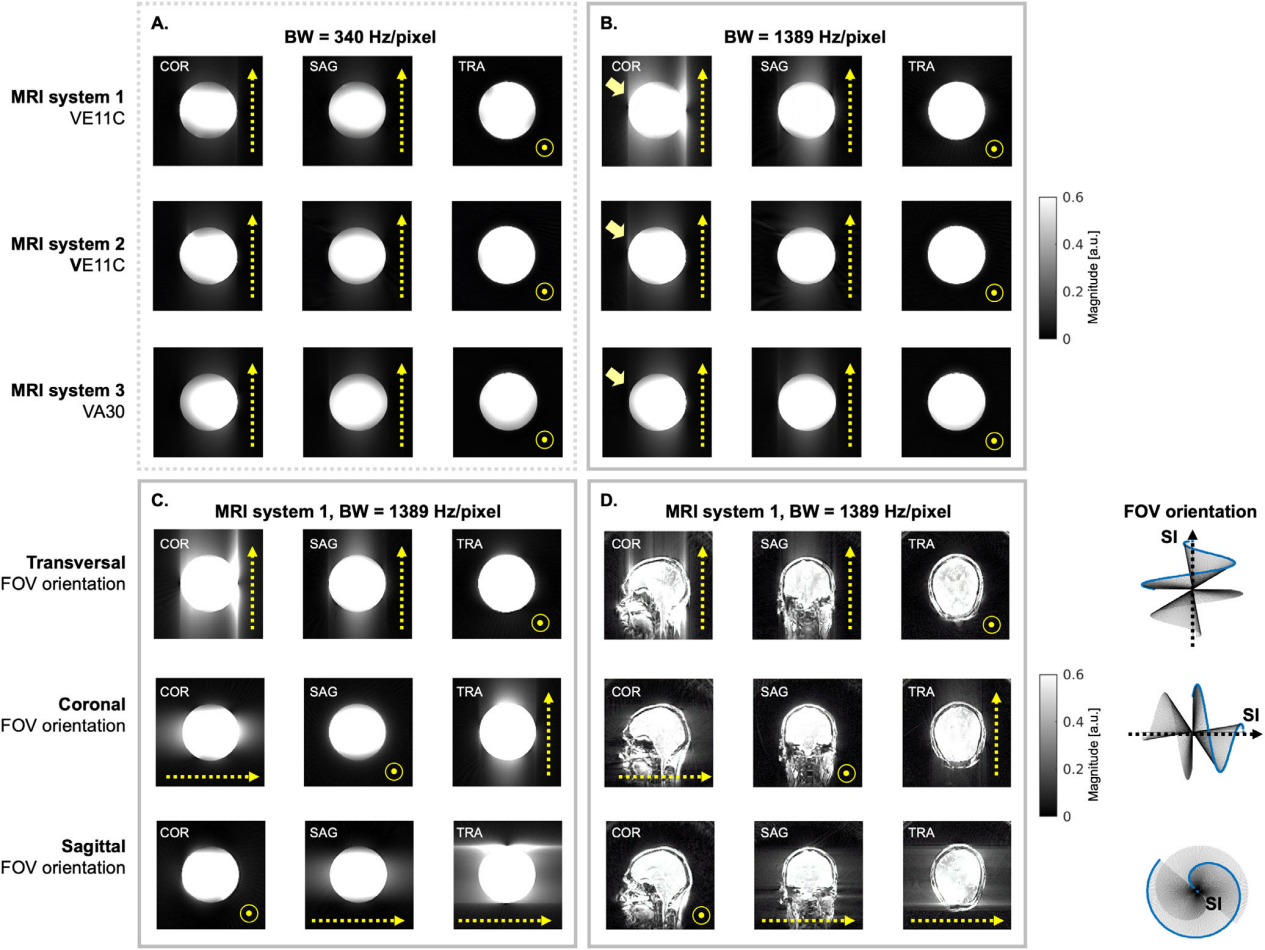
Phase‐induced image artifacts across different 3T MRI systems using different bandwidths and scan orientations. (A) A spherical salt‐water phantom was used to acquire bSSFP data using the original phyllotaxis trajectory on three 3T MRI systems (MAGNETOM Prisma, Siemens Healthineers, Forchheim, Germany) at two receiver bandwidths (BW). (B) At MRI system 1, image artifacts were pronounced for higher values of the receiver BW. (C) Images acquired with a higher bandwidth showed an artifact, which was dependent on the field‐of‐view (FOV) orientations using MRI system 1. (D) An in vivo brain scan was performed on the same scanner using the same protocol as in (C), and FOV‐orientation dependent image artifacts were observed. The dotted arrow indicates the direction of the superior–inferior (SI) spoke, indicating the orientation of the phyllotaxis trajectory.

The bSSFP data acquired with the original phyllotaxis trajectory in a transverse FOV orientation showed image artifacts that varied in severity across different 3T MRI systems and receiver bandwidths. These artifacts became more severe at higher receiver bandwidth (Figure 2A,B). Repeating the scan with a higher bandwidth and with different FOV orientations—in both the phantom and the brain—revealed that the artifact was predominantly aligned along the direction of the SI‐spoke, which is related to the geometric shape of the phyllotaxis trajectory with respect to the orientation of the SI‐spoke, compared to other orientations (Figure 2C,D). Notably, only one of the three MRI systems was severely affected.

**FIGURE 3:**
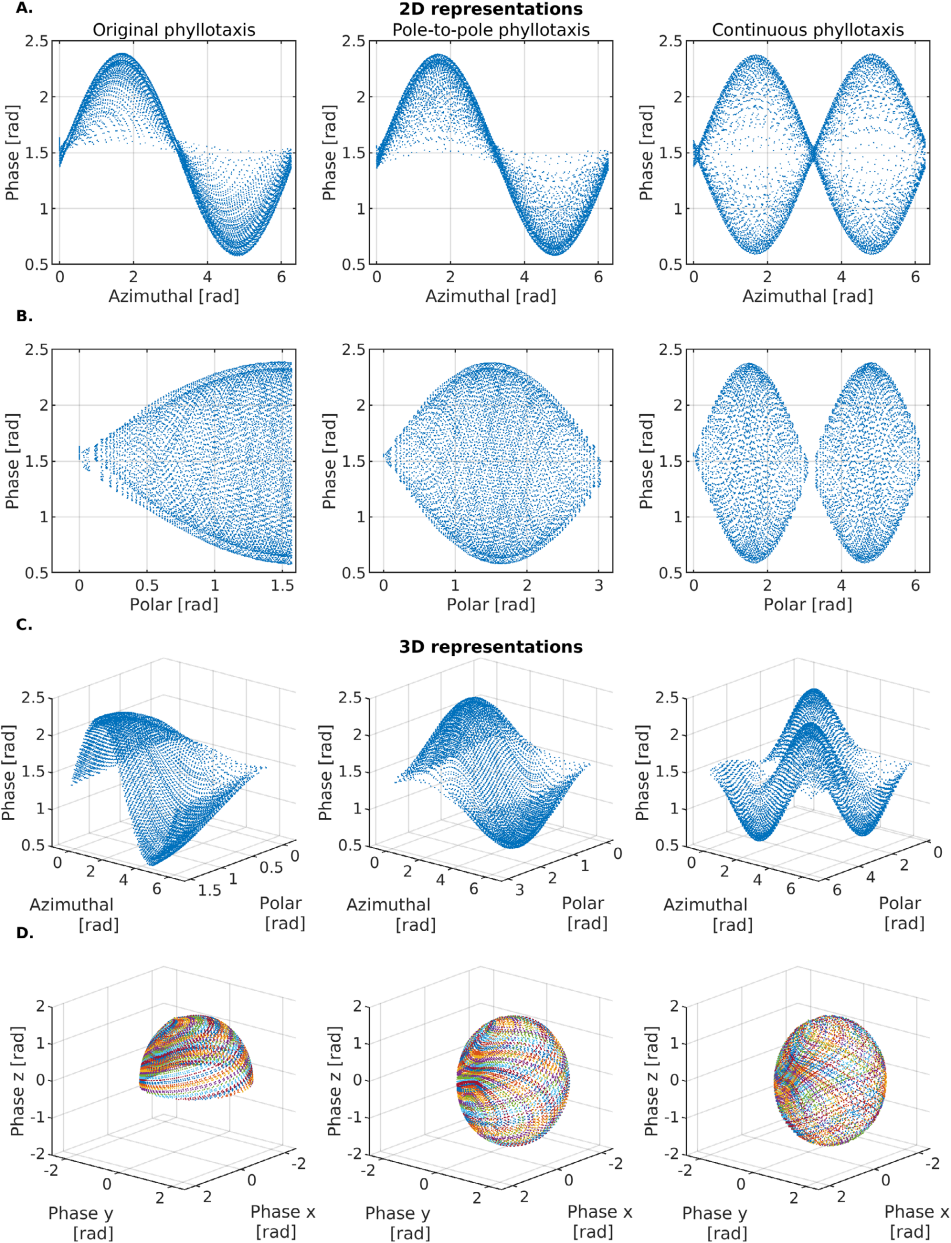
*K*‐space center phase variations. Phase variations for a single coil at the center of Phase variations from the 2nd through the 40th interleave are shown as a function of the (A) azimuthal and (B) polar angles of each spoke. (C) The same phase variations are plotted in 3D against the polar and azimuthal angles of each trajectory. (D) The phase variations are visualized in polar coordinates, where the radius represents the phase variation at the readout center. This representation maps phase variations in *k*‐space along the Cartesian *x*, *y*, and *z* coordinates of the scanner. Color coding, similar to Figure 1, distinguishes different interleaves.

The signal phase recorded at the *k*‐space center of bSSFP data revealed variations dependent on the polar and azimuthal angles (φ and θ), exhibiting a distinct periodic pattern (Figure 3). This pattern appeared consistent for all three 3D radial trajectories. However, a more intuitive representation of the data in spherical coordinates (Figure 3D) enabled spatial visualization of phase offsets relative to the physical scanner axes and showed that the phase was more evenly distributed across both hemispheres for the pole‐to‐pole and continuous trajectory. When comparing these spherical maps across different 3T MRI systems, there was a clear offset observed in one of the 3T MRI systems (Figure S1), indicating a system‐specific deviation along the *y*‐axis.

**FIGURE 4:**
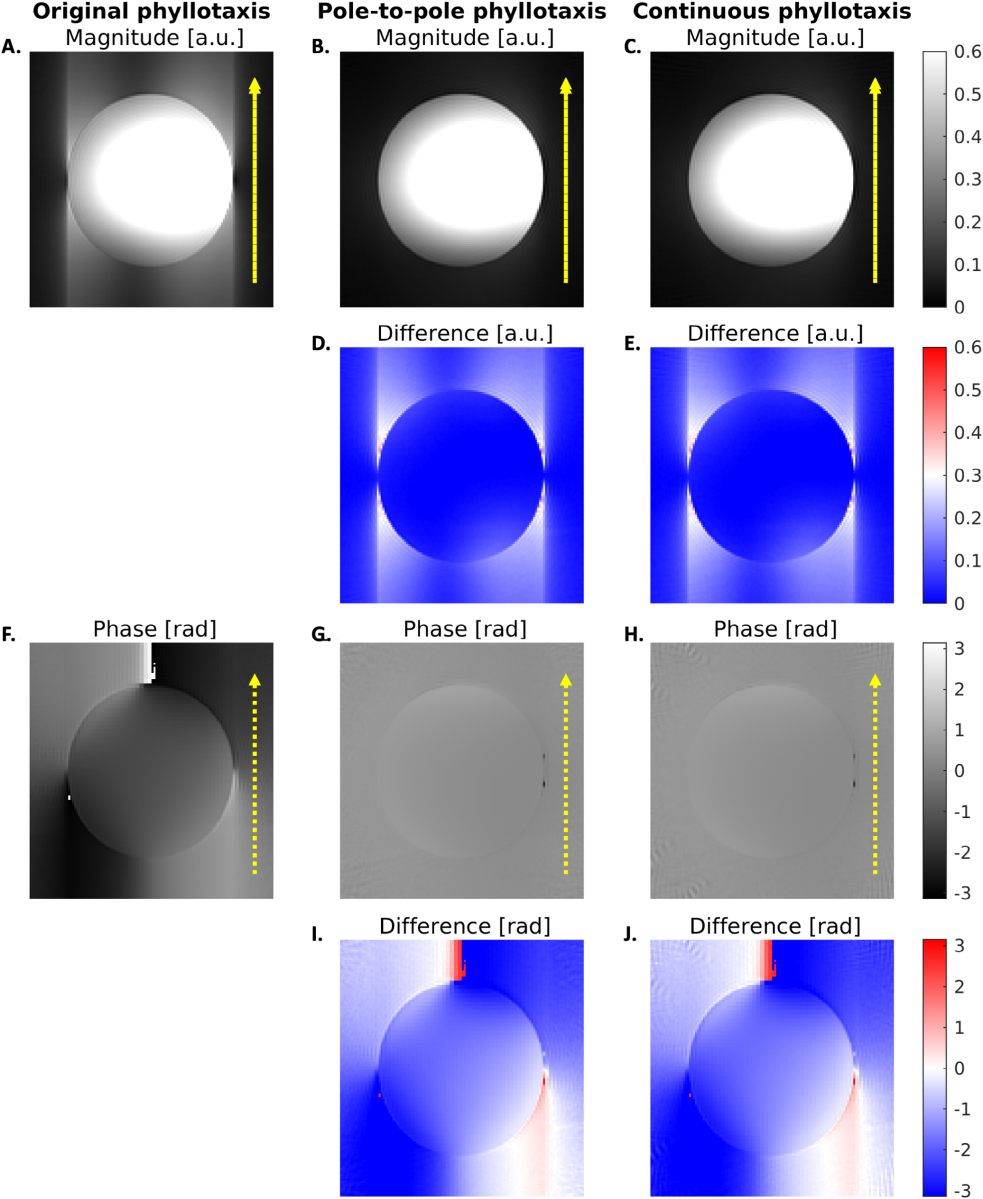
Image artifacts induced by different trajectories in a spherical salt‐water phantom. (A) The magnitude image from the original phyllotaxis trajectory shows artifacts along the SI‐spoke direction (dotted arrow), which are resolved using the (B) pole‐to‐pole and (C) continuous phyllotaxis trajectories. Magnitude difference maps between the original and pole‐to‐pole phyllotaxis (D) and between the original and continuous phyllotaxis (E). (F) The phase image from the original phyllotaxis trajectory shows an opposing offset along the SI‐spoke direction, which is absent in the imaging data using the (G) pole‐to‐pole and (H) continuous phyllotaxis trajectories. Phase difference maps between the original and pole‐to‐pole phyllotaxis (I) and between the original and continuous phyllotaxis (J).

In bSSFP magnitude images, artifacts were visible along the SI‐spoke direction when using the original phyllotaxis trajectory (Figure 4A), but not when using the pole‐to‐pole and continuous phyllotaxis trajectories (Figure 4B,C). Difference maps showed a magnitude image artifact reduction up to ∼50% (Figure 4D,E). Additionally, in the bSSFP phase images, phase offsets were observed in data acquired with the original phyllotaxis trajectory (Figure 4F). These phase offsets were absent when using the pole‐to‐pole and continuous trajectories, where the reconstructed phase image was close to zero (Figure 4G,H). No further improvement was observed when using the continuous phyllotaxis over the pole‐to‐pole phyllotaxis.

**FIGURE 5:**
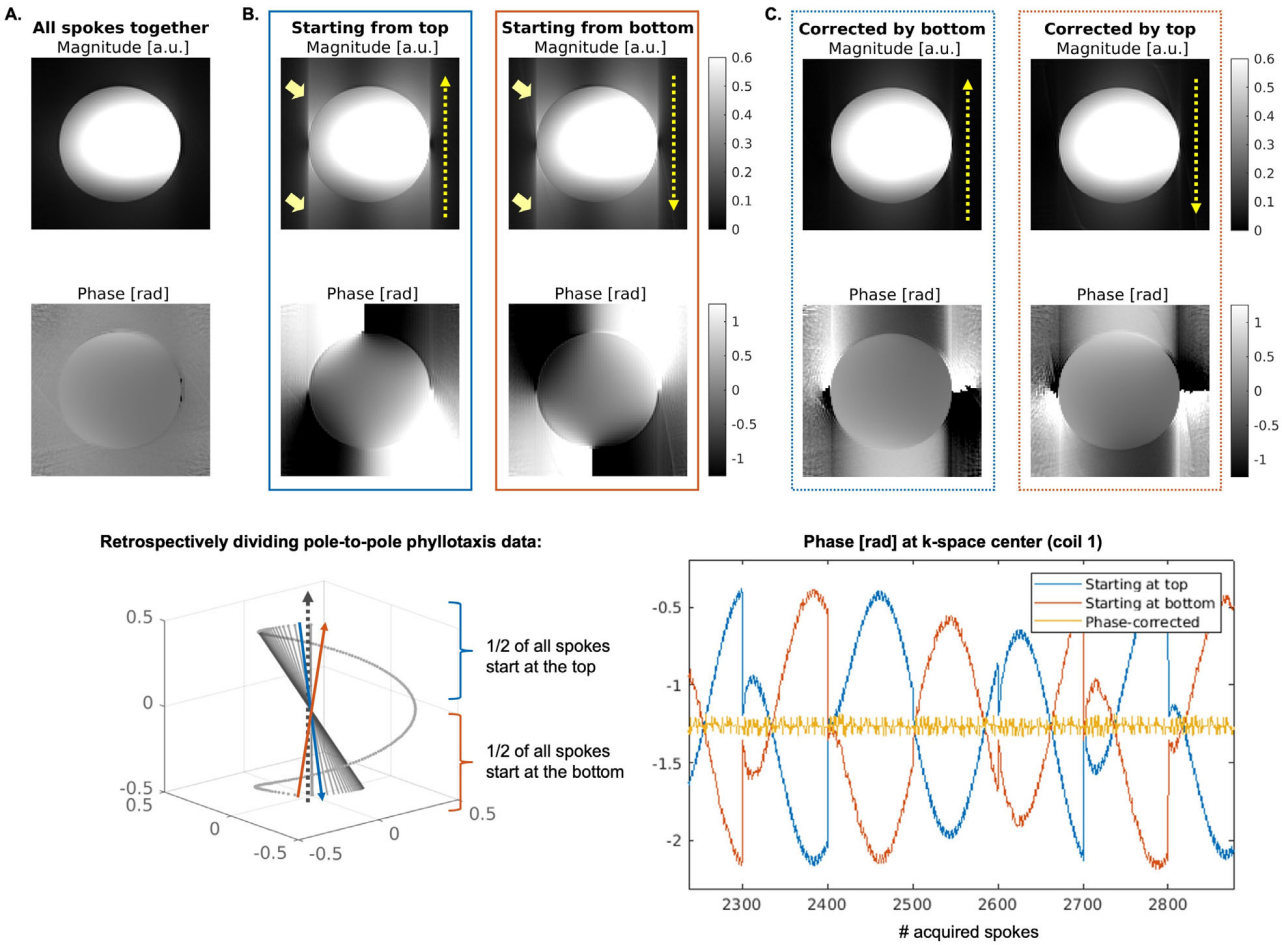
Phase correction. Magnitude and phase images reconstructed from data acquired using the pole‐to‐pole trajectory. (A) Using all spokes for image reconstruction does not show image artifacts in the SI‐direction as present in images reconstructed from data acquired using the original trajectory. (B) When only spokes that originate from the upper hemisphere (blue) and lower hemisphere (red) are used for image reconstruction, image artifacts reappear. Note that this represents image artifacts similar to using the original phyllotaxis trajectory. (C) When applying the proposed phase correction based on opposing spokes, the image artifact disappears.

Dividing the pole‐to‐pole dataset into two subsets reproduced the image artifact observed with the original phyllotaxis trajectory (Figure 5A,B). The phase exhibited an inverted pattern between the subsets. Applying the proposed spoke‐wise phase correction, mitigated the artifact and restored image quality comparable to the full pole‐to‐pole dataset (Figure 5C,D). A similar effect was observed with the gradient delay correction (Figure S5). In contrast, trajectory corrections based on the first order GIRF, that is the correction of linear eddy current effects on the gradients, did not eliminate the artifacts (Figure S2). Repeating each spoke of the pole‐to‐pole trajectory four times did not alter the artifacts or image quality between repetitions (Figure S6). Difference maps between images reconstructed from repeated spokes showed slightly higher deviations at the beginning of each interleave, with the continuous trajectory exhibiting the smallest differences (Figure S6E).

**FIGURE 6:**
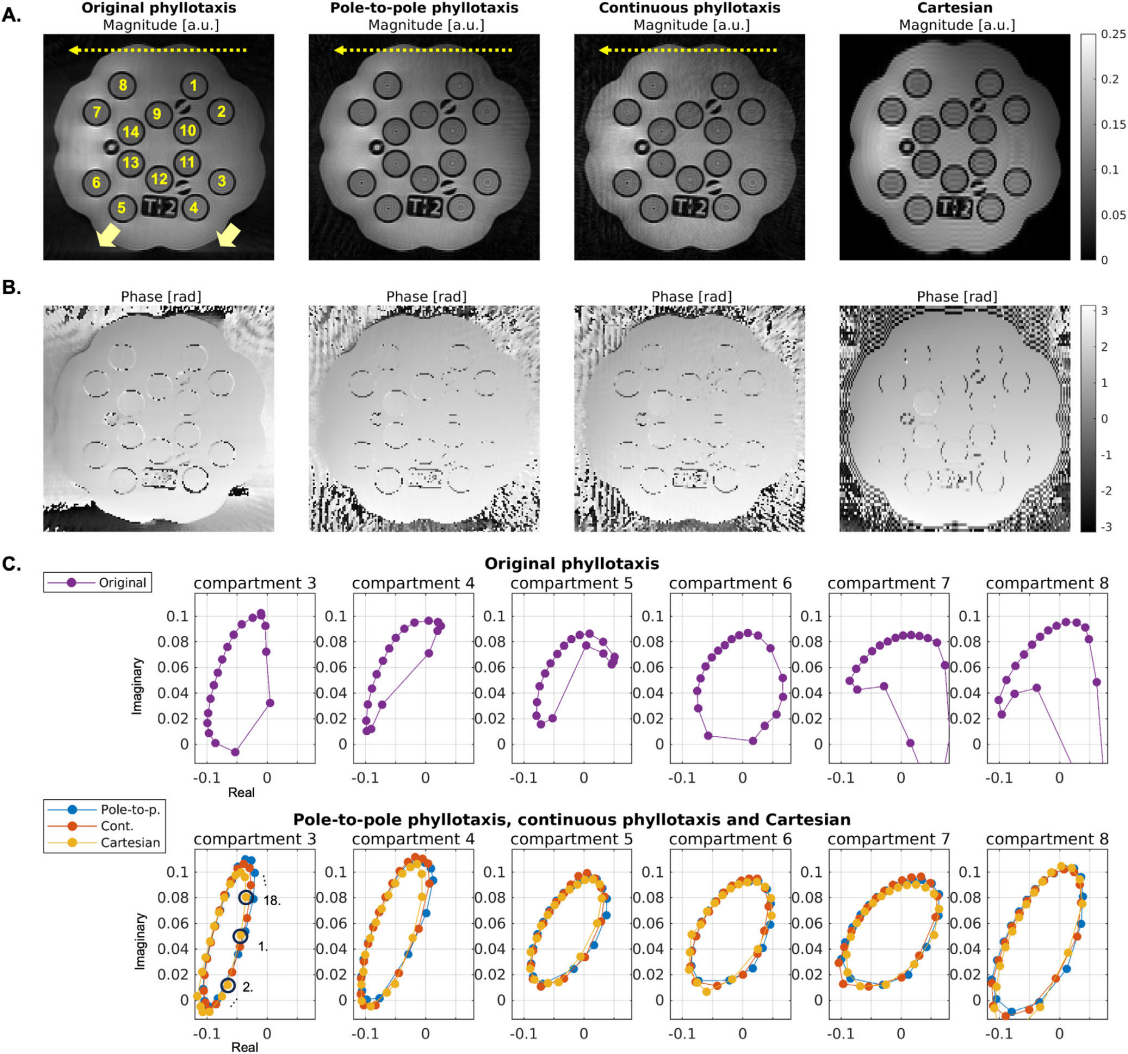
Phase‐cycled bSSFP scan of the MRI system phantom. (A) Magnitude images for the 180° RF phase increment across all radial trajectories, compared to a Cartesian acquisition and (B) corresponding phase images, contain signal smearing along the direction of the SI‐spoke. Mean signal values in six phantom compartments (C) plotted in the complex plane (real vs. imaginary) for the original phyllotaxis trajectory and for the two new radial trajectories and the Cartesian scan. The bSSFP profiles (1.–18. as indicated in the lower plots) derived from data acquired using original phyllotaxis trajectory show deviations in the expected elliptical shape with respect to the new trajectories and the Cartesian acquisition.

**FIGURE 7:**
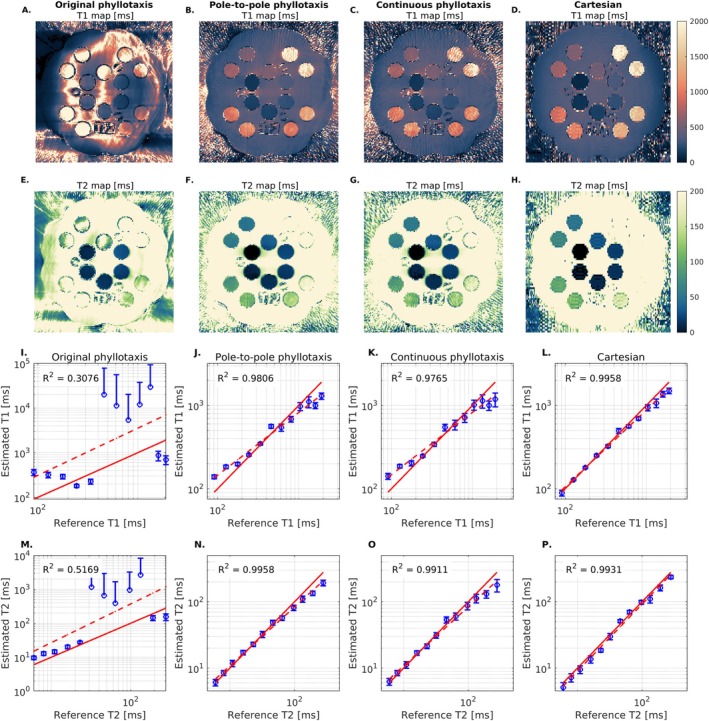
T1 and T2 maps of the MRI system phantom estimated using ORACLE. (A–D) T1 maps reconstructed from the original phyllotaxis, pole‐to‐pole phyllotaxis, continuous phyllotaxis, and Cartesian trajectories. (E–H) Corresponding T2 maps. The maps derived from data acquired with the original phyllotaxis trajectory show spatial deviations in T1 and T2 values. (I–L) Mean and standard deviation of T1 values within each compartment's ROI, plotted against reference values. (M–P) Mean and standard deviation of T2 values within each compartment's ROI, plotted against reference values. T1 and T2 values derived from data acquired with the original phyllotaxis trajectory do not match the references values, which is the case for the new trajectories and the Cartesian trajectory.

Phase‐cycled bSSFP scans in the standardized 136 mini system phantom MRI system phantom produced magnitude and phase images (Figure 6A,B) with artifacts along the SI‐spoke direction, similar to those observed in the spherical phantom. Elliptical [50] bSSFP profiles were observed in the compartments of the phantom for data reconstructed from acquisitions using the novel radial trajectories and the Cartesian trajectory but were distorted in the original phyllotaxis trajectory (Figure 6C). These distortions translated into visible artifacts in the resulting T1 and T2 maps (Figure 7A–H).

Quantitative maps derived from pole‐to‐pole phyllotaxis, continuous phyllotaxis, and Cartesian acquisitions closely matched reference phantom values (Figure 7I–P), while those from the original phyllotaxis trajectory showed significant discrepancies. The agreement between estimated and reference T1 values across the phantom vials were: *R* ^2^ = 0.3076 and RMSE = 10 971 ms (original phyllotaxis), *R* ^2^ = 0.9806 and RMSE = 261 ms (pole‐to‐pole phyllotaxis), *R* ^2^ = 0.9765 and RMSE = 280 ms (continuous phyllotaxis), and *R* ^2^ = 0.9958 and RMSE = 161 ms (Cartesian). For T2 these values were: *R* ^2^ = 0.5169 and RMSE = 863 ms (original phyllotaxis), *R* ^2^ = 0.9958 and RMSE = 31 ms (pole‐to‐pole phyllotaxis), *R* ^2^ = 0.9911 and RMSE = 34 ms (continuous phyllotaxis), and *R* ^2^ = 0.9931 and RMSE = 16 ms (Cartesian).

**FIGURE 8:**
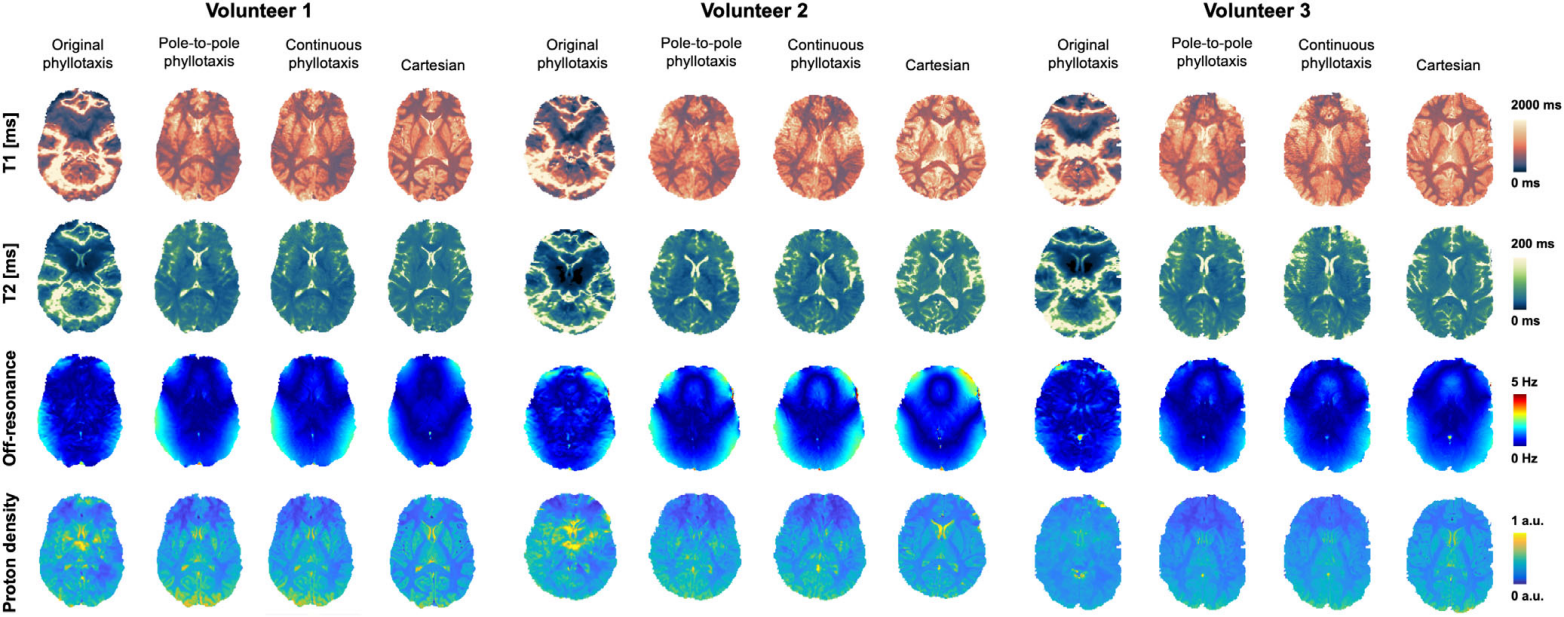
Sagittal and transversal views of in vivo brain scans. T1, T2, off‐resonance and proton density maps for all three subjects using the original phyllotaxis, pole‐to‐pole phyllotaxis, continuous phyllotaxis, and Cartesian trajectories. Variations in T1 and T2 maps derived from scans acquired using original phyllotaxis trajectory are not visible in maps derived from scans using acquired using pole‐to‐pole and the continuous phyllotaxis trajectory and the Cartesian trajectory.

In brain bSSFP scans, T1, T2, off‐resonance, and proton density maps reconstructed from data acquired using the pole‐to‐pole phyllotaxis, continuous phyllotaxis, and Cartesian trajectories appeared visually consistent within each subject (Figure 8). In contrast, parametric maps from the original phyllotaxis trajectory exhibited widespread T1 and T2 deviations across the brain in all volunteer scans.

**FIGURE 9:**
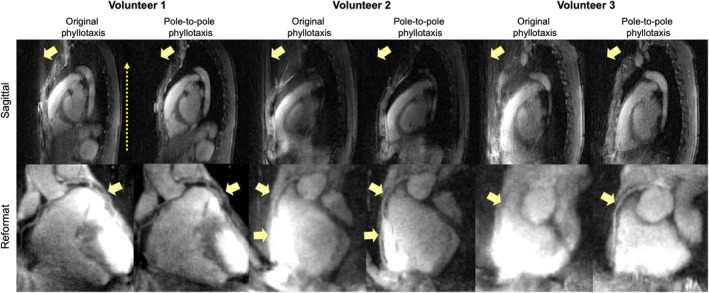
Free‐breathing whole‐heart MRI at 3T. Free‐breathing ECG‐triggered GRE experiments were performed in three healthy volunteers. Cardiac images are shown in sagittal, coronal, and reformatted views to emphasize coronary artery structures. Signal smearing is absent in the images reconstructed from data acquired with the pole‐to‐pole trajectory and result in sharper coronary artery visualization.

In cardiac data, the original phyllotaxis trajectory introduced artifacts across all three healthy volunteers (Figure 9A–C). These artifacts were absent when using the pole‐to‐pole phyllotaxis trajectory.

## 4 Discussion

This study demonstrated that the proposed pole‐to‐pole and continuous 3D radial phyllotaxis trajectories effectively mitigate magnitude and phase‐related artifacts by acquiring radial spokes from opposite directions throughout the scan without the need for additional correction methods. The new 3D radial trajectory designs resulted in substantial improvements in image quality in phantoms, in the brain, and in the heart of healthy volunteers when compared with the original spiral phyllotaxis trajectory, regardless of using a bSSFP or a GRE sequence. In addition, quantitative T1 and T2 mapping was improved in both phantom and brain data.

Phantom experiments suggested a directional dependency of an 3D radial imaging artifact, which rotated with the FOV, and a scanner dependency of the artifact, as the artifact strength varied between scanners and was stronger at higher bandwidths. The SI‐spoke direction was consistently the predominant axis of artifact formation, even when the FOV was rotated, which may be attributed to the lack of opposing spokes in the *z*‐direction in the original phyllotaxis trajectory.

Additionally, phase offsets that varied with both polar and azimuthal angles were observed suggesting a trajectory dependency, which have been observed before [14, 53]. As the *k*‐space center phase should be independent of spoke orientation, this indicates the presence of gradient delay effects caused by system imperfections. Therefore, the spherical representation of the phase variation in *k*‐space may be used to test different MRI scanners and to illustrate gradient related effects. For example, amongst the three MRI scanners used, the graphical representation showed a clear phase‐offset in one of the scanner axes of one of the scanners (Figure S1).

Images reconstructed from data acquired with the new trajectory designs did not show significant artifacts. This is due to the inclusion of nearly opposing spokes, supported by the findings of retrospectively dividing the pole‐to‐pole phyllotaxis dataset into two subsets resembling the original phyllotaxis design: phase compensation between nearly opposing spoke pairs effectively suppressed the artifacts, underscoring the value of directional symmetry in radial sampling. The applied first order GIRF correction did not resolve the artifact (Figure S2), by not compensating for a global phase offset. Although subtle, the continuous trajectory exhibited the smallest phase variations at the beginning of each interleave (Figure S6E). In addition, using a trajectory with large *k*‐space jumps can introduce artifacts, whereas changing the position relative to the isocenter has no noticeable effect (Figure S5).

The new trajectories reduced T1 and T2 quantification errors compared to the original phyllotaxis trajectory. However, T1 overestimation (for T1 values < 200 ms), and small residual errors and radial streaks remained visible in the T1 and T2 maps when using the new trajectories compared to the Cartesian scan, especially in the peripheral regions of the phantom. This may be due to aliasing, or streaking, of the signal from the liquid surrounding the vials into the vials. Alternatively, it may indicate the presence of residual eddy current effects. This is because all radial trajectories were implemented with a fixed number of interleaves, resulting in larger jumps between spokes in the pole‐to‐pole and continuous trajectory designs. In phase‐cycled bSSFP, eddy currents can introduce phase accumulation and residual gradient moments, which violate the assumption of perfect gradient balancing within each TR. This affects both, the steady state, including the expected linear phase increments, as well as the effective trajectory and nearly opposing spokes might not completely eliminate this effect. The underestimation of high T1 values (∼> 2000 ms) across all trajectories is attributed to B1^+^ inhomogeneity in peripheral regions, as also evident in the T1 maps of the phantom's background medium.

The reconstructed spoiled GRE scans of the heart showed a reduction in artifacts caused by similar phase inconsistencies when using the pole‐to‐pole trajectory. To maintain the same acquisition window length in the triggered acquisition, the number of spokes per interleave was kept constant for both trajectories. As a result, the pole‐to‐pole trajectory exhibited larger angular jumps between spokes. These acquisitions also demonstrate the robustness of the new trajectory designs for accelerated acquisitions. Although undersampling introduces larger angular gaps between opposing spokes, the compensating effect of near‐opposing spokes remained. By retrospectively creating even larger gaps between spokes (Figure S3), it could be shown that the compensation effects can still be preserved in linear reconstructions up to an acceleration factor of 20.

Although phase errors can stem from multiple sources—including gradient timing mismatches, system‐specific hardware delays, bandwidth, and resolution—our findings show that adding opposing spokes to the trajectory design can reduce their impact. This is similar to the concept of using a few opposing spokes in the form of a pre‐scan, as has been suggested before, showing that gradient delays [14, 28] can be corrected by estimating signal shifts from them [27]. Similarly, in bSTAR [54, 55], an ultra‐short echo time (UTE) technique [43, 56, 57], center‐out and center‐in half radial spokes are sampled from all directions in *k*‐space. Another, well‐known effect can be observed in Cartesian echo‐planar imaging (EPI) trajectories, where Nyquist ghost corrections address phase inconsistencies between odd and even *k*‐space lines [58] caused by gradient delays, eddy currents, or system timing errors, which manifest as ghost images. Similarly, our proposed phase correction mitigates global phase inconsistencies between opposing spokes, ensuring that system imperfections do not propagate into artifacts, particularly in phase‐sensitive applications. Further investigation with dedicated hardware setups [26] would be needed to fully characterize the underlying sources of phase variation in 3D radial MRI and refine trajectory designs accordingly.

Beyond phase‐cycled bSSFP, the improved phase consistency of the new trajectories suggests broader applicability in MRI methods that rely on signal phase information, such as quantitative susceptibility mapping [59], flow encoding [60, 61], or MR elastography [62].

The results presented here showed that some MRI systems may produce artifacts that compromise the value and utility of 3D radial acquisitions, but which can be overcome with the proposed designs without any further corrections. These trajectory modifications could be extended to other applications that currently employ the original 3D radial phyllotaxis, such as FISS [11, 63], bSSFP or spoiled GRE sequences targeting moving organs. Other 3D radial designs, such as the Archimedean spiral [17, 18], and 3D radial MRI in general, may similarly benefit from pole‐to‐pole adaptations, particularly in instances where the MRI system exhibits imperfections.

Despite certain limitations, the proposed pole‐to‐pole and continuous phyllotaxis trajectories provide a simple, robust method for mitigating trajectory‐induced phase artifacts in 3D radial MRI. They offer improved accuracy in both qualitative and quantitative imaging without requiring further corrections. The main strengths of non‐Cartesian imaging lie in applications such as cardiac triggering, motion binning, and accelerated imaging in combination with compressed sensing, which can be used in combination with the new trajectory designs even at higher acceleration factors (Figure S3). These scenarios particularly benefit from the flexibility and robustness of radial sampling, which is challenging to achieve in practice with Cartesian trajectories. Future work could explore targeted correction strategies that build on these designs, as well as their application across other MRI sequences. These trajectories hold promise for advancing the fidelity and reliability of radial MRI acquisitions across a range of clinical and research applications.

## 5 Conclusion

The proposed 3D radial phyllotaxis trajectory designs removed image artifacts associated with phase inconsistencies. The inclusion of opposing spokes in 3D radial sampling prevented signal smearing and improved the visualization of anatomical structures in the heart and in the brain, and improved the reliability of phase‐dependent quantitative imaging applications.

## Funding

This work was supported by Schweizerischer Nationalfonds zur Förderung der Wissenschaftlichen Forschung (PCEFP2_194296).

## Conflicts of Interest

Davide Piccini is an employee of Siemens Healthineers. Li Feng is co‐inventor of a patent on the GRASP and XD‐GRASP MRI techniques and a patent on the MRSIGMA technique.

## Supporting information


**Figure S1:** The phase offset at the *k*‐space center for the first coil, and the 2nd to 40th interleave, is displayed in spherical coordinates as shown in Figure 3. In MRI system 1, the y‐direction exhibited an asymmetry in the spherical distribution, consistent across all FOV orientations. This asymmetry was absent in MRI system 2.
**Figure S2:** The GIRF acquired on MRI system 1 (A). Magnitude images of the bSSFP scans in the spherical salt‐water phantom using the original phyllotaxis trajectory (B) with a first order GIRF correction.
**Figure S3:** Retrospectively undersampled data (acceleration factors *R* = 8, *R* = 20) acquired with the pole‐to‐pole phyllotaxis trajectory and reconstructed using linear reconstruction (i.e., without using compressed sensing). (A) Reconstructed magnitude images and (B) reconstructed phase images with corresponding line plots along the phase image. (C) Despite the larger angles between each spoke and its closest opposing spokes due to retrospective undersampling, the compensatory properties of the trajectory remained unchanged, even at an acceleration factor of 20.
**Figure S4:** Acquisitions using the original phyllotaxis acquisition of a 180‐degree RF phase increment bSSFP scan in the spherical saltwater phantom, as described in the second experiment. The scans were taken at low (A) and high (B) receiver bandwidths (BW), which again indicates the dependency of the artifacts on the readout gradient strength, which is lower at a lower bandwidth. Repeating the experiment at a high BW with a randomized trajectory and the same number of spokes (C) reveals eddy current patterns. Repeating the (D) scans with the phantom shifted with respect to the isocenter shows no substantial change in artifact appearance.
**Figure S5:** Reconstructing all spokes of the pole‐to‐pole trajectory together results in (A), while adding an additional gradient delay correction results in (B). Reconstructing only the top half of the pole‐to‐pole trajectory (which “simulates” the original phyllotaxis sampling) results in (C)—with visual appearance of the described artifacts. Applying a gradient delay correction on this data results in (D), which leads to a correction of the artifact. Applying the gradient delay correction by applying solely the fitted slope (E) or intercept (F) of the phase fit, shows that general phase offset (the intercept) is important for the phase correction.
**Figure S6:** Experiment 2 was repeated with each spoke acquired four times consecutively (Rep 1–Rep 4), under the hypothesis that eddy current effects would diminish by the 4th repetition. Images were reconstructed separately for each repetition. To highlight differences, magnitude images from consecutive repetitions were divided (e.g., Rep 1/Rep 2, etc.), with the resulting difference plots shown in (A–C). Interestingly, differences were less pronounced between odd and even repetitions for scans with 180° phase increments. No substantial differences between the two trajectories were observed in these difference images, suggesting that eddy currents were not the dominant effect. As before, the SI spoke was discarded for reconstruction. (D) The *k*‐space center magnitude for all four repetitions is shown for spokes acquired at the beginning of the sequence, before steady state was reached (these were discarded during acquisition). Odd and even repetitions exhibit opposite effects, with a small residual difference that corresponds to the zig‐zag pattern in the phase seen in Figure 5. (E) Importantly, when averaging magnitude differences (Rep 1/Rep 3 and Rep 2/Rep 4) across all interleaves in steady state, the continuous trajectory shows the smallest deviation for the first spokes at the start of each interleave, that is immediately after the “jump” in *k*‐space. Since the first spoke (the SI spoke) is discarded during reconstruction, this explains why no substantial differences are observed between the two new trajectory designs in the final images.

## Data Availability

All raw data are available for download from https://zenodo.org/records/17428583, https://zenodo.org/records/18098352 and https://zenodo.org/records/18098464. The code for calculating and visualizing the phyllotaxis trajectories, as well as for reconstructing the 3D radial data with phase and gradient delay corrections, is available at: https://github.com/QIS‐MRI/3D_radial_phyllotaxis_pole‐to‐pole.git. This repository also includes compiled Pulseq .seq files to reproduce experiments 1–2.

## References

[1] C. Stehning , P. Börnert , K. Nehrke , H. Eggers , and M. Stuber , “Free‐Breathing Whole‐Heart Coronary MRA With 3D Radial SSFP and Self‐Navigated Image Reconstruction,” Magnetic Resonance in Medicine 54, no. 2 (2005): 476–480, 10.1002/mrm.20557. 16032682

[2] D. Piccini , A. Littmann , S. Nielles‐Vallespin , and M. O. Zenge , “Spiral Phyllotaxis: The Natural Way to Construct a 3D Radial Trajectory in MRI,” Magnetic Resonance in Medicine 66, no. 4 (2011): 1049–1056, 10.1002/mrm.22898. 21469185

[3] D. Piccini , L. Feng , G. Bonanno , et al., “Four‐Dimensional Respiratory Motion‐Resolved Whole Heart Coronary MR Angiography,” Magnetic Resonance in Medicine 77, no. 4 (2017): 1473–1484, 10.1002/mrm.26221. 27052418 PMC5040623

[4] M. Lustig , D. Donoho , and J. M. Pauly , “Sparse MRI: The Application of Compressed Sensing for Rapid MR Imaging,” Magnetic Resonance in Medicine 58, no. 6 (2007): 1182–1195, 10.1002/mrm.21391. 17969013

[5] L. Feng , S. Coppo , D. Piccini , et al., “5D Whole‐Heart Sparse MRI,” Magnetic Resonance in Medicine 79, no. 2 (2018): 826–838, 10.1002/mrm.26745. 28497486 PMC5681898

[6] J. A. M. Bastiaansen , R. B. van Heeswijk , M. Stuber , and D. Piccini , “Noncontrast Free‐Breathing Respiratory Self‐Navigated Coronary Artery Cardiovascular Magnetic Resonance Angiography at 3 T Using Lipid Insensitive Binomial Off‐Resonant Excitation (LIBRE),” Journal of Cardiovascular Magnetic Resonance 21, no. 1 (2019): 38, 10.1186/s12968-019-0543-6. 31291957 PMC6621993

[7] G. Bauman , N. G. Lee , Y. Tian , O. Bieri , and K. S. Nayak , “Submillimeter Lung MRI at 0.55 T Using Balanced Steady‐State Free Precession With Half‐Radial Dual‐Echo Readout (bSTAR),” Magnetic Resonance in Medicine 90, no. 5 (2023): 1949–1957, 10.1002/mrm.29757. 37317635

[8] G. Bauman , K. M. Johnson , L. C. Bell , et al., “Three‐Dimensional Pulmonary Perfusion MRI With Radial Ultrashort Echo Time and Spatial–Temporal Constrained Reconstruction,” Magnetic Resonance in Medicine 73, no. 2 (2015): 555–564, 10.1002/mrm.25158. 24604452 PMC4156934

[9] B. Kaltenbach , A. Roman , C. Polkowski , et al., “Free‐Breathing Dynamic Liver Examination Using a Radial 3D T1‐Weighted Gradient Echo Sequence With Moderate Undersampling for Patients With Limited Breath‐Holding Capacity,” European Journal of Radiology 86 (2017): 26–32, 10.1016/j.ejrad.2016.11.003. 28027757

[10] B. Franceschiello , L. Di Sopra , A. Minier , et al., “3‐Dimensional Magnetic Resonance Imaging of the Freely Moving Human Eye,” Progress in Neurobiology 194 (2020): 101885, 10.1016/j.pneurobio.2020.101885. 32653462

[11] J. A. M. Bastiaansen , D. Piccini , L. Di Sopra , et al., “Natively Fat‐Suppressed 5D Whole‐Heart MRI With a Radial Free‐Running Fast‐Interrupted Steady‐State (FISS) Sequence at 1.5T and 3T,” Magnetic Resonance in Medicine 83, no. 1 (2020): 45–55, 10.1002/mrm.27942. 31452244 PMC6778714

[12] A. L. C. Mackowiak , D. Piccini , R. B. van Heeswijk , R. Hullin , C. Gräni , and J. A. M. Bastiaansen , “Fat‐Free Noncontrast Whole‐Heart CMR With Fast and Power‐Optimized Off‐Resonant Water Excitation Pulses,” Journal of Cardiovascular Magnetic Resonance 26 (2024): 101096, 10.1016/j.jocmr.2024.101096. 39278414 PMC11616052

[13] N. Masala , J. A. M. Bastiaansen , L. Di Sopra , et al., “Free‐Running 5D Coronary MR Angiography at 1.5T Using LIBRE Water Excitation Pulses,” Magnetic Resonance in Medicine 84, no. 3 (2020): 1470–1485, 10.1002/mrm.28221. 32144824

[14] D. C. Peters , J. A. Derbyshire , and E. R. McVeigh , “Centering the Projection Reconstruction Trajectory: Reducing Gradient Delay Errors,” Magnetic Resonance in Medicine 50, no. 1 (2003): 1–6, 10.1002/mrm.10501. 12815671 PMC2034322

[15] A. Moussavi , M. Untenberger , M. Uecker , and J. Frahm , “Correction of Gradient‐Induced Phase Errors in Radial MRI,” Magnetic Resonance in Medicine 71, no. 1 (2014): 308–312, 10.1002/mrm.24643. 23440722

[16] J. H. Duyn , Y. Yang , J. A. Frank , and J. W. van der Veen , “Simple Correction Method Fork‐Space Trajectory Deviations in MRI,” Journal of Magnetic Resonance 132, no. 1 (1998): 150–153, 10.1006/jmre.1998.1396. 9615415

[17] S. T. S. Wong and M. S. Roos , “A Strategy for Sampling on a Sphere Applied to 3D Selective RF Pulse Design,” Magnetic Resonance in Medicine 32, no. 6 (1994): 778–784, 10.1002/mrm.1910320614. 7869901

[18] S. Nielles‐Vallespin , P. Spier , X. Bi , D. Li , and E. Müller , “Navigator‐Gated Whole Heart Coronary MRI: Comparison of 3D TrueFisp Cartesian and Radial Acquisitions,” (2006), 366, In: Proceedings of the 14th Annual Meeting of ISMRM, Seattle, Washington, USA.

[19] L. Braunstorfer , J. Romanowicz , A. J. Powell , et al., “Non‐Contrast Free‐Breathing Whole‐Heart 3D Cine Cardiovascular Magnetic Resonance With a Novel 3D Radial Leaf Trajectory,” Magnetic Resonance Imaging 94 (2022): 64–72, 10.1016/j.mri.2022.09.003. 36122675 PMC9631735

[20] L. Feng , R. Grimm , K. T. Block , et al., “Golden‐Angle Radial Sparse Parallel MRI: Combination of Compressed Sensing, Parallel Imaging, and Golden‐Angle Radial Sampling for Fast and Flexible Dynamic Volumetric MRI,” Magnetic Resonance in Medicine 72, no. 3 (2014): 707–717, 10.1002/mrm.24980. 24142845 PMC3991777

[21] A. Fyrdahl , K. Holst , K. Caidahl , M. Ugander , and A. Sigfridsson , “Generalization of Three‐Dimensional Golden‐Angle Radial Acquisition to Reduce Eddy Current Artifacts in bSSFP CMR Imaging,” Magnetic Resonance Materials in Physics, Biology and Medicine 34, no. 1 (2021): 109–118, 10.1007/s10334-020-00859-z. PMC7910232 32592094

[22] N. Scholand , P. Schaten , C. Graf , et al., “Rational Approximation of Golden Angles: Accelerated Reconstructions for Radial MRI,” Magnetic Resonance in Medicine 93 (2024): 51, 10.1002/mrm.30247. 39250418 PMC12034029

[23] S. J. Vannesjo , N. N. Graedel , L. Kasper , et al., “Image Reconstruction Using a Gradient Impulse Response Model for Trajectory Prediction,” Magnetic Resonance in Medicine 76, no. 1 (2016): 45–58, 10.1002/mrm.25841. 26211410

[24] M. Stich , T. Wech , A. Slawig , et al., “Gradient Waveform Pre‐Emphasis Based on the Gradient System Transfer Function,” Magnetic Resonance in Medicine 80, no. 4 (2018): 1521–1532, 10.1002/mrm.27147. 29479736

[25] P. Daudé , T. Troalen , A. L. C. Mackowiak , et al., “Trajectory Correction Enables Free‐Running Chemical Shift Encoded Imaging for Accurate Cardiac Proton‐Density Fat Fraction Quantification at 3T,” Journal of Cardiovascular Magnetic Resonance 26, no. 2 (2024): 101048, 10.1016/j.jocmr.2024.101048. 38878970 PMC11269917

[26] B. E. Dietrich , D. O. Brunner , B. J. Wilm , et al., “A Field Camera for MR Sequence Monitoring and System Analysis,” Magnetic Resonance in Medicine 75, no. 4 (2016): 1831–1840, 10.1002/mrm.25770. 25975352

[27] K. T. U. M. Block , “Simple Method for Adaptive Gradient‐Delay Compensation in Radial MRI,” (2011), 2816, In: Proceedings of the 14th Annual Meeting of ISMRM, Seattle, Washington, USA.

[28] M. Untenberger , Z. Tan , D. Voit , et al., “Advances in Real‐Time Phase‐Contrast Flow MRI Using Asymmetric Radial Gradient Echoes,” Magnetic Resonance in Medicine 75, no. 5 (2016): 1901–1908, 10.1002/mrm.25696. 26096085

[29] S. Rosenzweig , H. C. M. Holme , and M. Uecker , “Simple Auto‐Calibrated Gradient Delay Estimation From Few Spokes Using Radial Intersections (RING),” Magnetic Resonance in Medicine 81, no. 3 (2019): 1898–1906, 10.1002/mrm.27506. 30489652

[30] S. Smith David , “WEB Self‐Calibrated Gradient Delay Correction for Golden Angle Radial MRI,” (2014), In: Proceedings of the 14th Annual Meeting of ISMRM, Seattle, Washington, USA.

[31] G. M. C. Rossi , A. L. C. Mackowiak , B. C. Açikgöz , et al., “SPARCQ: A New Approach for Fat Fraction Mapping Using Asymmetries in the Phase‐Cycled Balanced SSFP Signal Profile,” Magnetic Resonance in Medicine 90, no. 6 (2023): 2348–2361, 10.1002/mrm.29813. 37496187

[32] Y. Shcherbakova , C. A. T. van den Berg , C. T. W. Moonen , and L. W. Bartels , “PLANET: An Ellipse Fitting Approach for Simultaneous T_1_ and T_2_ Mapping Using Phase‐Cycled Balanced Steady‐State Free Precession,” Magnetic Resonance in Medicine 79, no. 2 (2018): 711–722, 10.1002/mrm.26717. 28543430 PMC5811804

[33] O. Bieri , C. Weidensteiner , and C. Ganter , “Robust T 2 Estimation With Balanced Steady State Free Precession,” Magnetic Resonance in Medicine 91, no. 6 (2024): 2257–2265, 10.1002/mrm.30037. 38411351

[34] L. Feng , “Golden‐Angle Radial MRI: Basics, Advances, and Applications,” Journal of Magnetic Resonance Imaging 56, no. 1 (2022): 45–62. 35396897 10.1002/jmri.28187 PMC9189059

[35] D. Nguyen and O. Bieri , “Motion‐Insensitive Rapid Configuration Relaxometry,” Magnetic Resonance in Medicine 78, no. 2 (2017): 518–526, 10.1002/mrm.26384. 27605508

[36] Y. Shcherbakova , C. A. T. van den Berg , C. T. W. Moonen , and L. W. Bartels , “On the Accuracy and Precision of PLANET for Multiparametric MRI Using Phase‐Cycled bSSFP Imaging,” Magnetic Resonance in Medicine 81, no. 3 (2019): 1534–1552, 10.1002/mrm.27491. 30303562 PMC6585657

[37] N. M. J. Plähn , Y. Safarkhanlo , B. C. Açikgöz , et al., “ORACLE: An Analytical Approach for T1, T2, Proton Density, and Off‐Resonance Mapping With Phase‐Cycled Balanced Steady‐State Free Precession,” Magnetic Resonance in Medicine 93 (2024): 1657, 10.1002/mrm.30388. 39710877

[38] R. Heule , J. Bause , O. Pusterla , and K. Scheffler , “Multi‐Parametric Artificial Neural Network Fitting of Phase‐Cycled Balanced Steady‐State Free Precession Data,” Magnetic Resonance in Medicine 84, no. 6 (2020): 2981–2993, 10.1002/mrm.28325. 32479661

[39] B. C. Acikgoz , C. Sainz Martinez , A. L. C. Mackowiak , et al., “Quantitative Susceptibility Mapping in the Human Brain at 7T With Phase‐Cycled Balanced SSFP,” Magnetic Resonance in Medicine 94 (2025): 30571, 10.1002/mrm.30571. 40457642

[40] T. Bruijnen , B. Stemkens , C. A. T. van den Berg , and R. H. N. Tijssen , “Prospective GIRF‐Based RF Phase Cycling to Reduce Eddy Current‐Induced Steady‐State Disruption in bSSFP Imaging,” Magnetic Resonance in Medicine 84, no. 1 (2020): 115–127, 10.1002/mrm.28097. 31755580 PMC7154723

[41] O. Bieri , M. Markl , and K. Scheffler , “Analysis and Compensation of Eddy Currents in Balanced SSFP,” Magnetic Resonance in Medicine 54, no. 1 (2005): 129–137, 10.1002/mrm.20527. 15968648

[42] Y. Yang , J. Hair , J. Yerly , et al., “Quiescent Frame, Contrast‐Enhanced Coronary Magnetic Resonance Angiography Reconstructed Using Limited Number of Physiologic Frames From 5D Free‐Running Acquisitions,” Magnetic Resonance Imaging 113 (2024): 110209, 10.1016/j.mri.2024.07.008. 38972471 PMC11390311

[43] J. Delacoste , J. Chaptinel , C. Beigelman‐Aubry , D. Piccini , A. Sauty , and M. Stuber , “A Double Echo Ultra Short Echo Time (UTE) Acquisition for Respiratory Motion‐Suppressed High Resolution Imaging of the Lung,” Magnetic Resonance in Medicine 79, no. 4 (2018): 2297–2305, 10.1002/mrm.26891. 28856720

[44] S. J. Vannesjo , M. Haeberlin , L. Kasper , et al., “Gradient System Characterization by Impulse Response Measurements With a Dynamic Field Camera,” Magnetic Resonance in Medicine 69, no. 2 (2013): 583–593, 10.1002/mrm.24263. 22499483

[45] J. A. M. Bastiaansen and M. Stuber , “Flexible Water Excitation for Fat‐Free MRI at 3T Using Lipid Insensitive Binomial Off‐Resonant RF Excitation (LIBRE) Pulses,” Magnetic Resonance in Medicine 79, no. 6 (2018): 3007–3017, 10.1002/mrm.26965. 29159947

[46] P. Kim , D. Wendell , E. A. Park , H. Kim , W. Lee , and W. G. Rehwald , “A Fat Suppressed Adiabatic T2‐Preparation Module for 3T,” Journal of Cardiovascular Magnetic Resonance 15 (2013): E52, 10.1186/1532-429X-15-S1-E52.

[47] A. Schwarzl , “gpuNUFFT,” https://github.com/andyschwarzl/gpuNUFFT.

[48] L. Feng , Q. Wen , C. Huang , A. Tong , F. Liu , and H. Chandarana , “GRASP‐Pro: imProving GRASP DCE‐MRI Through Self‐Calibrating Subspace‐Modeling and Contrast Phase Automation,” Magnetic Resonance in Medicine 83, no. 1 (2020): 94–108, 10.1002/mrm.27903. 31400028 PMC6778712

[49] D. O. Walsh , A. F. Gmitro , and M. W. Marcellin , “Adaptive Reconstruction of Phased Array MR Imagery,” Magnetic Resonance in Medicine 43, no. 5 (2000): 682–690. 10800033 10.1002/(sici)1522-2594(200005)43:5<682::aid-mrm10>3.0.co;2-g

[50] Q. Xiang and M. N. Hoff , “Banding Artifact Removal for bSSFP Imaging With an Elliptical Signal Model,” Magnetic Resonance in Medicine 71, no. 3 (2014): 927–933, 10.1002/mrm.25098. 24436006

[51] M. Tagliabue , A. Mackowiak , L. Feng , E. Peper , and J. Bastiaansen , “Improving Coronary Assessment in 3D Radial Cardiac MRI Using Self‐Gating With K‐Space Center Filtering, Motion Binning, and CS Reconstruction,” (2025), Proceedings of the International Society for Magnetic Resonance.

[52] A. Etienne , R. M. Botnar , A. M. C. van Muiswinkel , P. Boesiger , W. J. Manning , and M. Stuber , ““Soap‐Bubble” Visualization and Quantitative Analysis of 3D Coronary Magnetic Resonance Angiograms,” Magnetic Resonance in Medicine 48, no. 4 (2002): 658–666, 10.1002/mrm.10253. 12353283

[53] L. Di Sopra , D. Piccini , S. Coppo , M. Stuber , and J. Yerly , “An Automated Approach to Fully Self‐Gated Free‐Running Cardiac and Respiratory Motion‐Resolved 5D Whole‐Heart MRI,” Magnetic Resonance in Medicine 82, no. 6 (2019): 2118–2132, 10.1002/mrm.27898. 31321816

[54] N. G. Lee , G. Bauman , O. Bieri , and K. S. Nayak , “Replication of the bSTAR Sequence and Open‐Source Implementation,” Magnetic Resonance in Medicine 91, no. 4 (2024): 1464–1477, 10.1002/mrm.29947. 38044680 PMC10872427

[55] O. Bieri , O. Pusterla , and G. Bauman , “Free‐Breathing Half‐Radial Dual‐Echo Balanced Steady‐State Free Precession Thoracic Imaging With Wobbling Archimedean Spiral Pole Trajectories,” Zeitschrift für Medizinische Physik 33, no. 2 (2023): 220–229, 10.1016/j.zemedi.2022.01.003. 35190223 PMC10311259

[56] X. Shen , A. C. Özen , H. Monsivais , et al., “High‐Resolution 3D Ultra‐Short Echo Time MRI With Rosette k‐Space Pattern for Brain Iron Content Mapping,” Journal of Trace Elements in Medicine and Biology 77 (2023): 127146, 10.1016/j.jtemb.2023.127146. 36871432 PMC10107748

[57] B. Chen , Y. Zhao , X. Cheng , et al., “Three‐Dimensional Ultrashort Echo Time Cones (3D UTE‐Cones) Magnetic Resonance Imaging of Entheses and Tendons,” Magnetic Resonance Imaging 49 (2018): 4–9, 10.1016/j.mri.2017.12.034. 29309823

[58] N. Chen and A. M. Wyrwicz , “Removal of EPI Nyquist Ghost Artifacts With Two‐Dimensional Phase Correction,” Magnetic Resonance in Medicine 51, no. 6 (2004): 1247–1253, 10.1002/mrm.20097. 15170846

[59] Y. Wang and T. Liu , “Quantitative Susceptibility Mapping (QSM): Decoding MRI Data for a Tissue Magnetic Biomarker,” Magnetic Resonance in Medicine 73, no. 1 (2015): 82–101, 10.1002/mrm.25358. 25044035 PMC4297605

[60] M. Markl , P. J. Kilner , and T. Ebbers , “Comprehensive 4D Velocity Mapping of the Heart and Great Vessels by Cardiovascular Magnetic Resonance,” Journal of Cardiovascular Magnetic Resonance 13 (2011): 7. 21235751 10.1186/1532-429X-13-7 PMC3025879

[61] E. S. Peper , P. van Ooij , B. Jung , A. Huber , C. Gräni , and J. A. M. Bastiaansen , “Advances in Machine Learning Applications for Cardiovascular 4D Flow MRI,” Frontiers in Cardiovascular Medicine 9 (2022): 9, 10.3389/fcvm.2022.1052068. PMC9780299 36568555

[62] A. Manduca , T. E. Oliphant , M. A. Dresner , et al., “Magnetic Resonance Elastography: Non‐Invasive Mapping of Tissue Elasticity,” Medical Image Analysis 5, no. 4 (2001): 237–254, 10.1016/S1361-8415(00)00039-6. 11731304

[63] M. B. L. Falcão , A. L. C. Mackowiak , G. M. C. Rossi , et al., “Combined Free‐Running Four‐Dimensional Anatomical and Flow Magnetic Resonance Imaging With Native Contrast Using Synchronization of Neighboring Acquisitions by Physiological Signals,” Journal of Cardiovascular Magnetic Resonance 26, no. 1 (2024): 101006, 10.1016/j.jocmr.2024.101006. 38309581 PMC11211232

