# Supplementary material for: Pole‐To‐Pole 3D Radial Trajectory Designs Improve Image Quality and Quantitative Parametric Mapping in the Brain and Heart
Source: Magn Reson Med. 2026 Jan 4;95(5):2627–43. doi: 10.1002/mrm.70237 (PMC12962215; doi:10.1002/mrm.70237)
Supplement: Supplementary file 1 — Figure S1: The phase offset at the k‐space center for the first coil, and the 2nd to 40th interleave, is displayed in spherical coordinates as shown in Figure 3. In MRI system 1, the y‐direction exhibited an asymmetry in the spherical distribution, consistent across all FOV orientations. This asymmetry was absent in MRI system 2. Figure S2: The GIRF acquired on MRI system 1 (A). Magnitude images of the bSSFP scans in the spherical salt‐water phantom using the original phyllotaxis trajectory (B) with a first order GIRF correction. Figure S3: Retrospectively undersampled data (acceleration factors R = 8, R = 20) acquired with the pole‐to‐pole phyllotaxis trajectory and reconstructed using linear reconstruction (i.e., without using compressed sensing). (A) Reconstructed magnitude images and (B) reconstructed phase images with corresponding line plots along the phase image. (C) Despite the larger angles between each spoke and its closest opposing spokes due to retrospective undersampling, the compensatory properties of the trajectory remained unchanged, even at an acceleration factor of 20. Figure S4: Acquisitions using the original phyllotaxis acquisition of a 180‐degree RF phase increment bSSFP scan in the spherical saltwater phantom, as described in the second experiment. The scans were taken at low (A) and high (B) receiver bandwidths (BW), which again indicates the dependency of the artifacts on the readout gradient strength, which is lower at a lower bandwidth. Repeating the experiment at a high BW with a randomized trajectory and the same number of spokes (C) reveals eddy current patterns. Repeating the (D) scans with the phantom shifted with respect to the isocenter shows no substantial change in artifact appearance. Figure S5: Reconstructing all spokes of the pole‐to‐pole trajectory together results in (A), while adding an additional gradient delay correction results in (B). Reconstructing only the top half of the pole‐to‐pole trajectory (which “simulates” the [file MRM-95-2627-s001.docx]

**Supporting Information Figures**

**
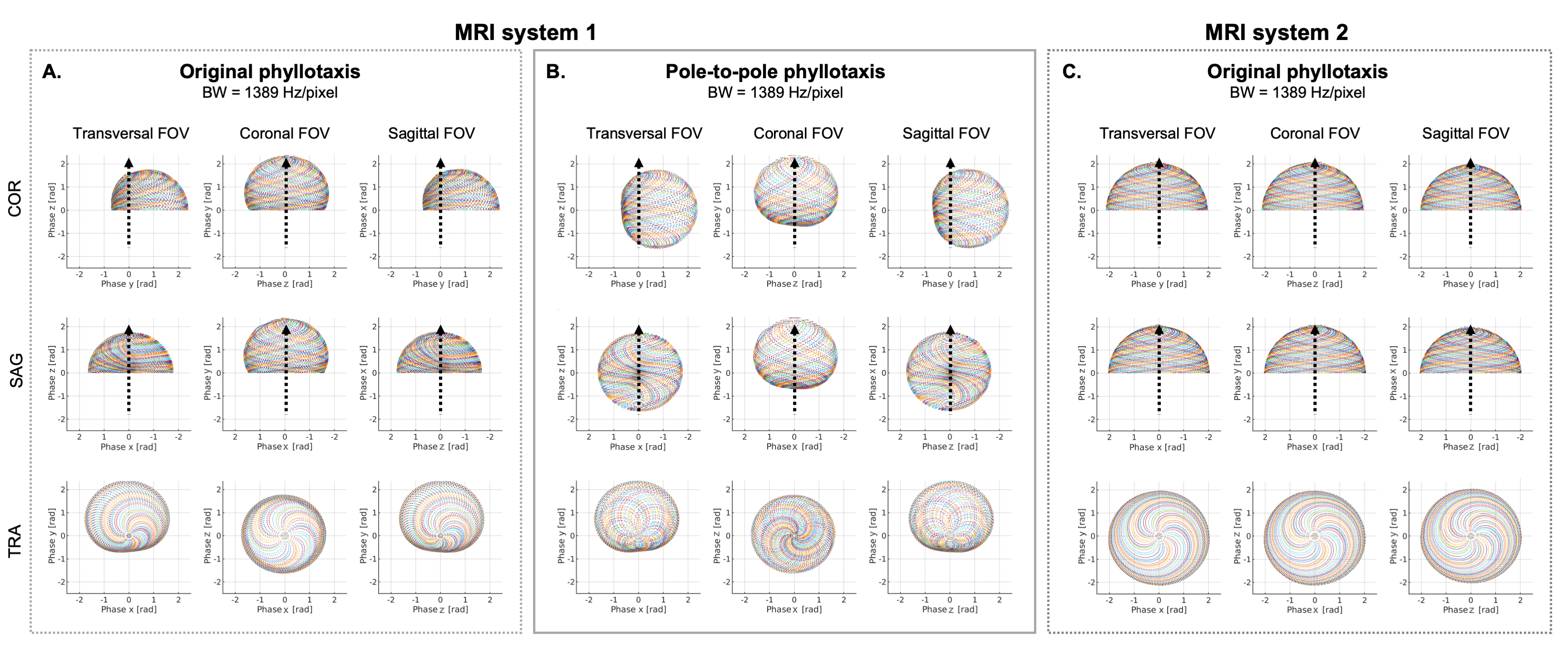
**

**Supporting Information Figure S1:** The phase offset at the k-space center for the first coil, and the 2nd to 40th interleave, is displayed in spherical coordinates as shown in Figure 3. In MRI system 1, the y-direction exhibited an asymmetry in the spherical distribution, consistent across all FOV orientations. This asymmetry was absent in MRI system 2.


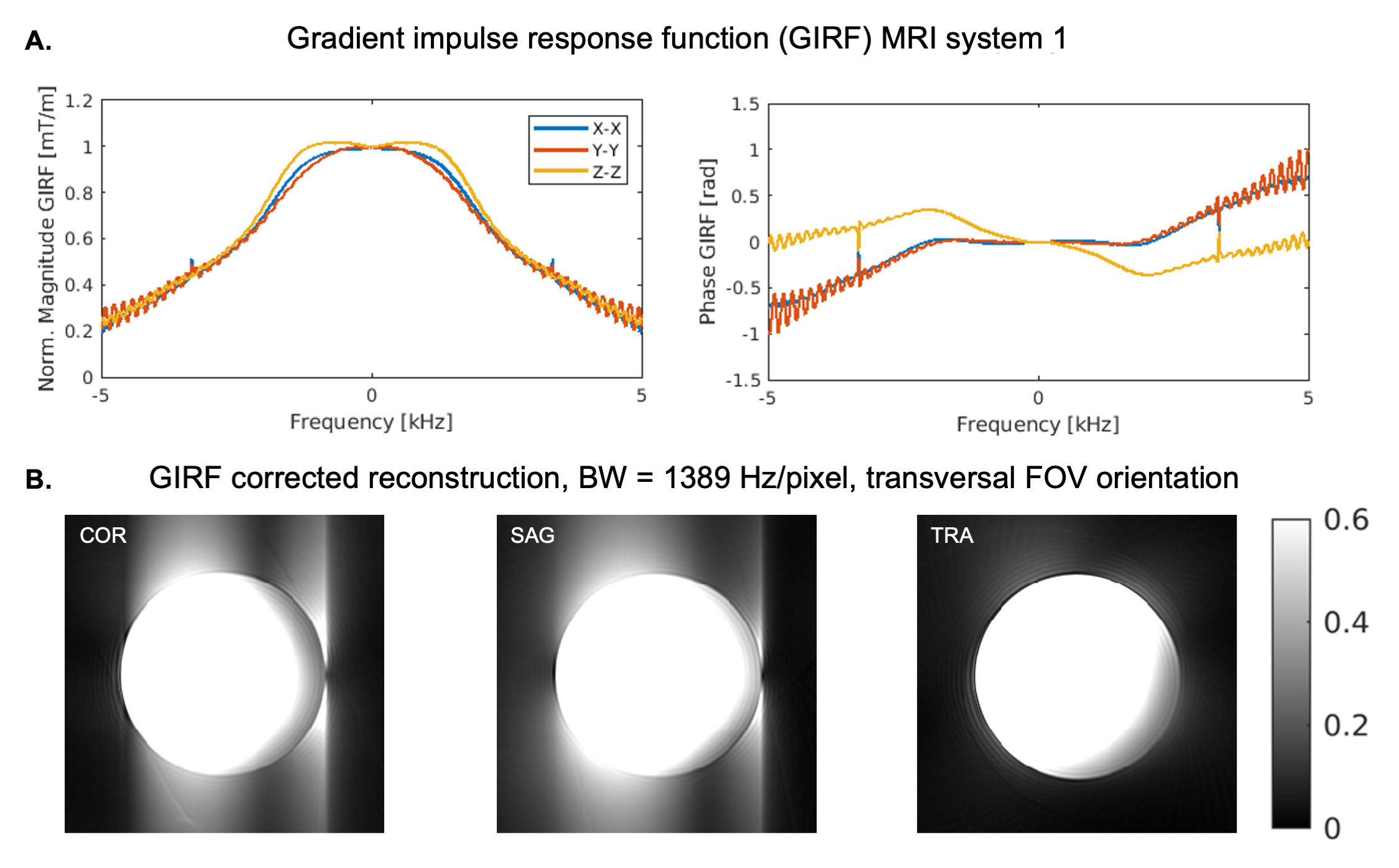


**Supporting Information Figure S2:** The GIRF acquired on MRI system 1 (A.). Magnitude images of the bSSFP scans in the spherical salt-water phantom using the original phyllotaxis trajectory (B.) with a first order GIRF correction.


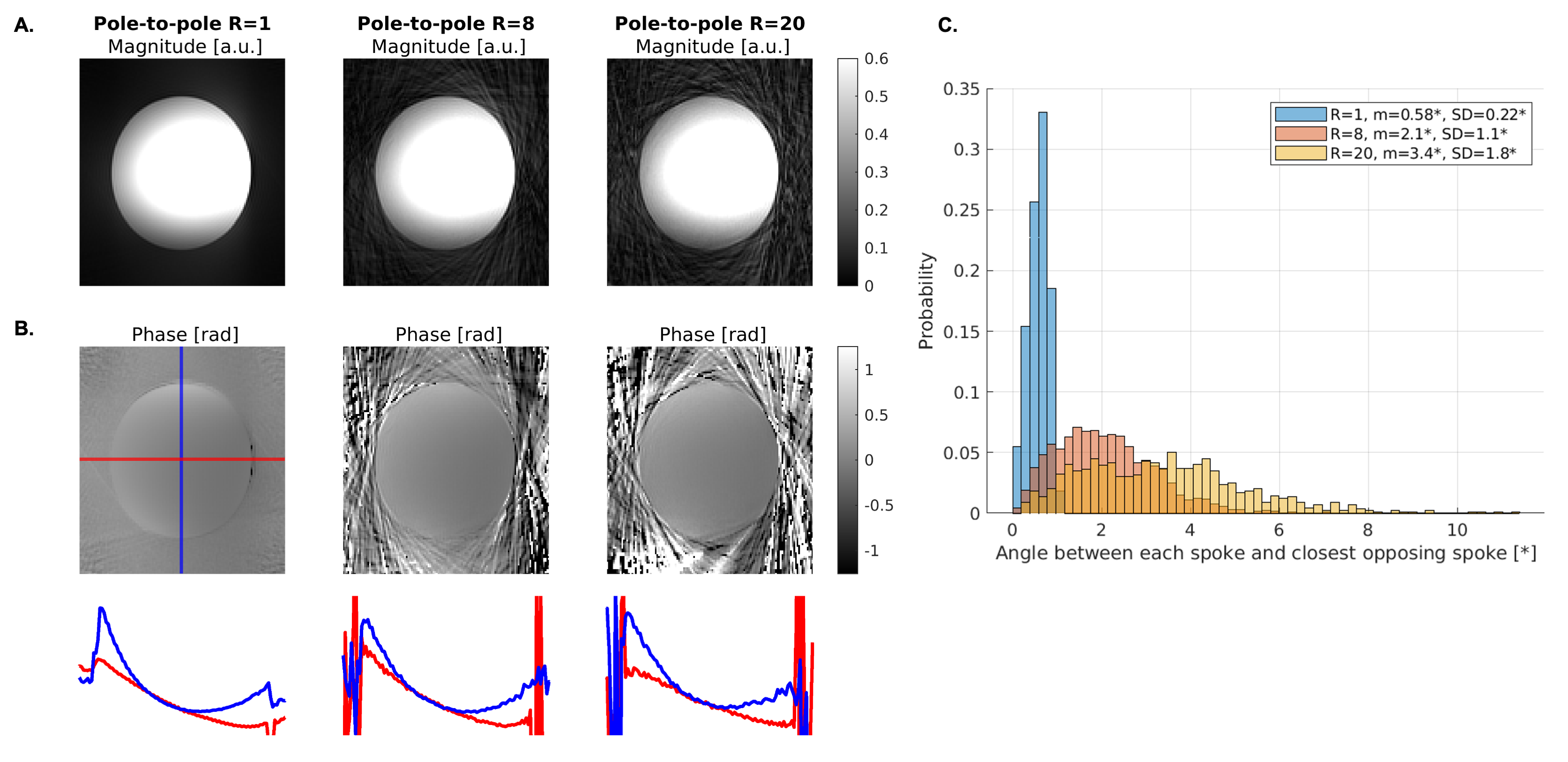


**Supporting Information Figure S3:** Retrospectively undersampled data (acceleration factors R=8, R=20) acquired with the pole-to-pole phyllotaxis trajectory and reconstructed using linear reconstruction (i.e., without using compressed sensing). (A.) Reconstructed magnitude images and (B.) reconstructed phase images with corresponding line plots along the phase image. (C.) Despite the larger angles between each spoke and its closest opposing spokes due to retrospective undersampling, the compensatory properties of the trajectory remained unchanged, even at an acceleration factor of 20.


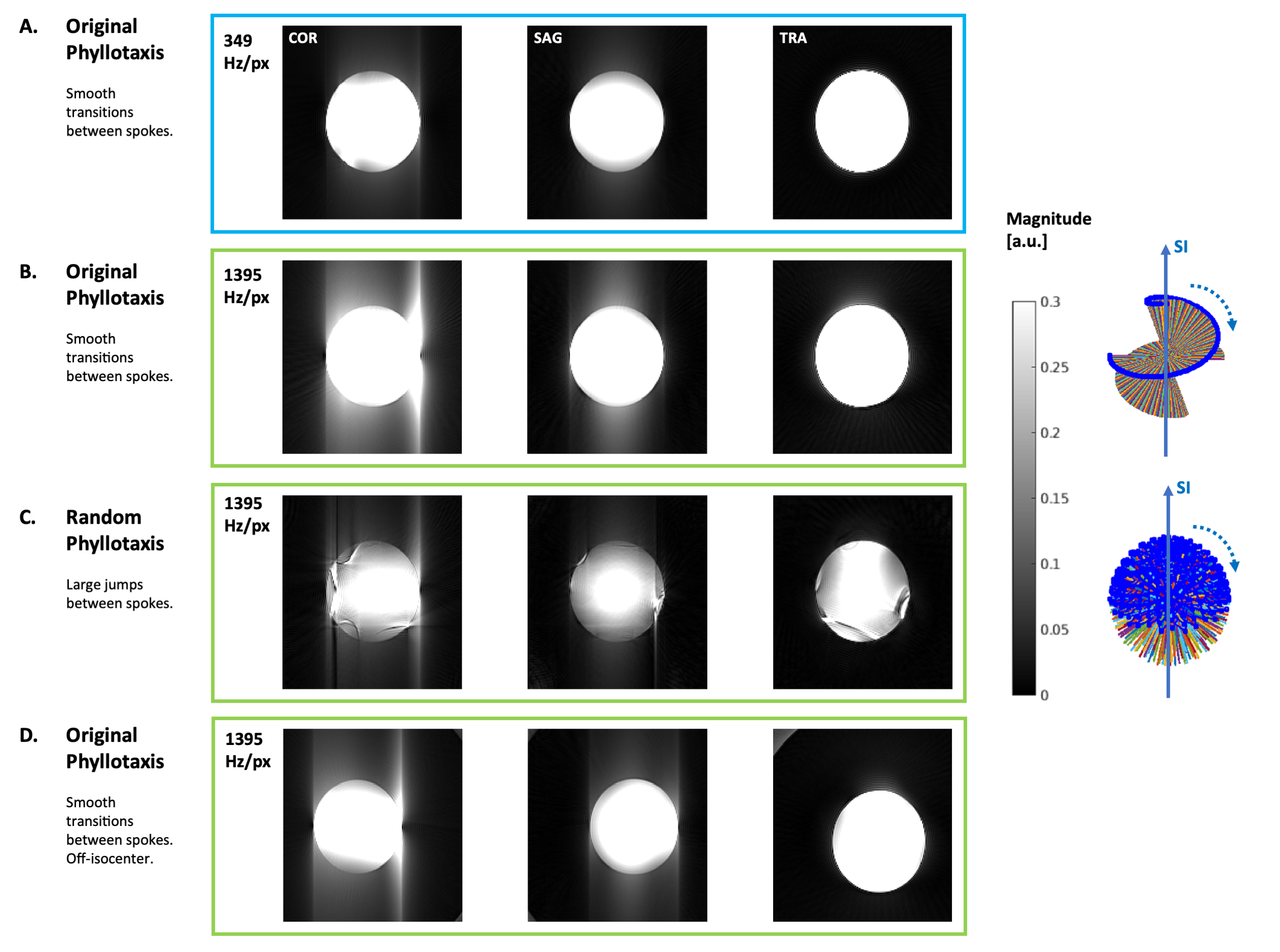


**Supporting Information Figure S4:** Acquisitions using the original phyllotaxis acquisition of a 180-degree RF phase increment bSSFP scan in the spherical saltwater phantom, as described in the second experiment. The scans were taken at low (A.) and high (B.) receiver bandwidths (BW), which again indicates the dependency of the artifacts on the readout gradient strength, which is lower at a lower bandwidth. Repeating the experiment at a high BW with a randomized trajectory and the same number of spokes (C.) reveals eddy current patterns^44^. Repeating the (D.) scans with the phantom shifted with respect to the isocenter shows no substantial change in artifact appearance.


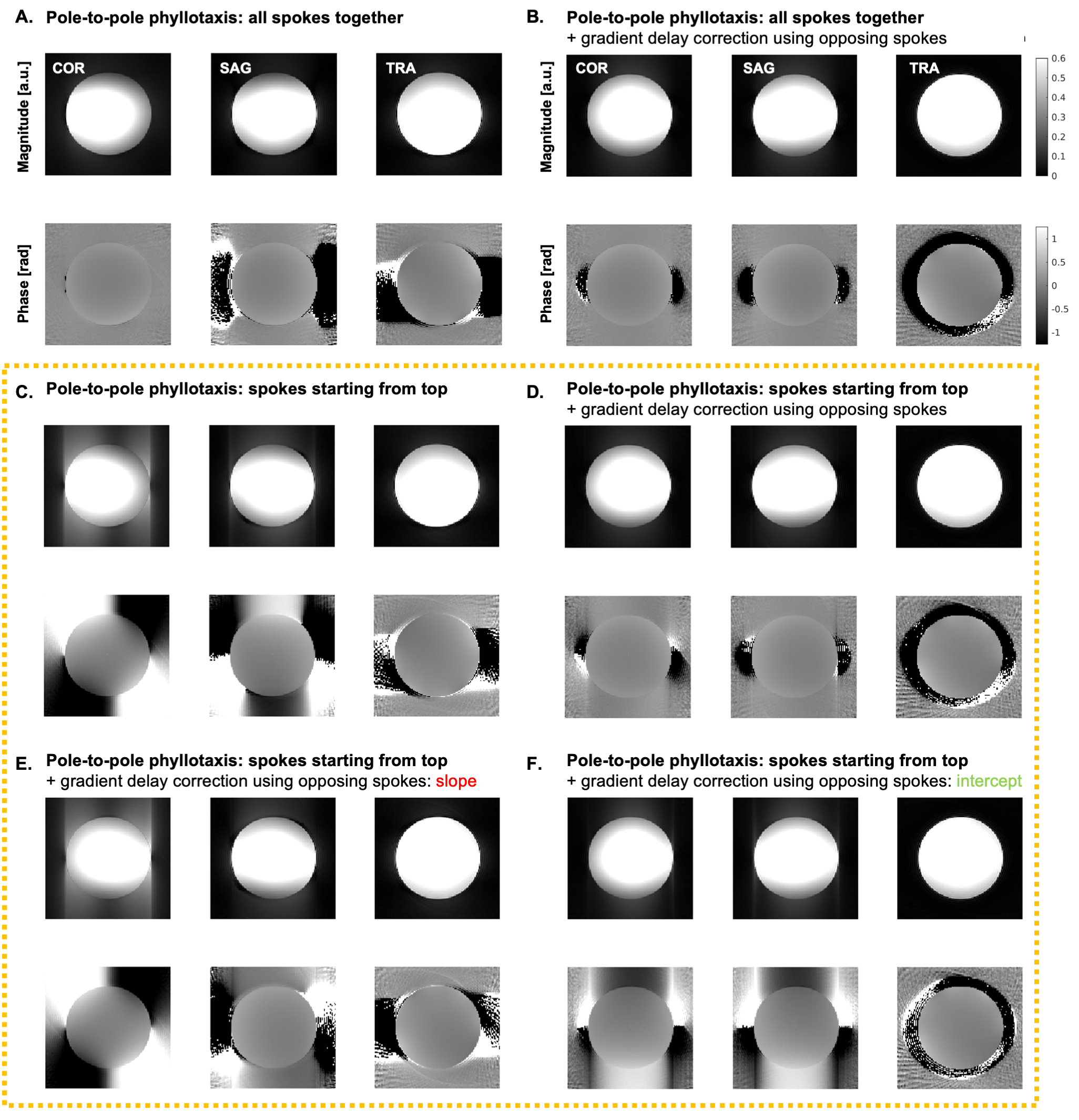


**Supporting Information Figure S5:** Reconstructing all spokes of the pole-to-pole trajectory together results in (A.), while adding an additional gradient delay correction results in (B.). Reconstructing only the top half of the pole-to-pole trajectory (which ‘simulates’ the original phyllotaxis sampling) results in (C.) - with visual appearance of the described artefacts. Applying a gradient delay correction on this data results in (D.), which leads to a correction of the artefact. Applying the gradient delay correction by applying solely the fitted slope (E.) or intercept (F.) of the phase fit, shows that general phase offset (the intercept) is important for the phase correction.


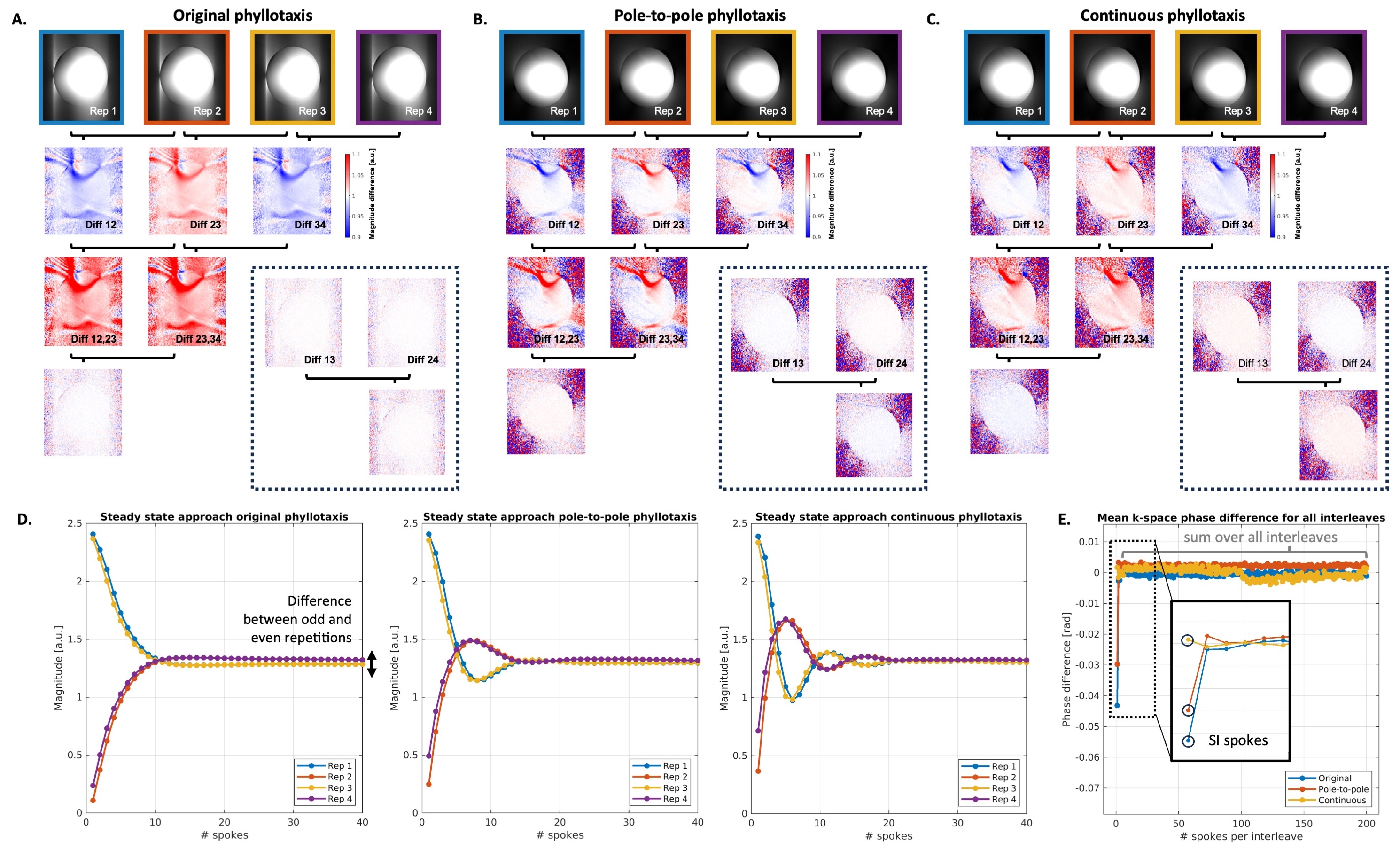


**Supporting Information Figure S6:** Experiment 2 was repeated with each spoke acquired four times consecutively (Rep 1–Rep 4), under the hypothesis that eddy current effects would diminish by the 4th repetition. Images were reconstructed separately for each repetition. To highlight differences, magnitude images from consecutive repetitions were divided (e.g., Rep 1 / Rep 2, etc.), with the resulting difference plots shown in (A.-C.). Interestingly, differences were less pronounced between odd and even repetitions for scans with 180° phase increments. No substantial differences between the two trajectories were observed in these difference images, suggesting that eddy currents were not the dominant effect. As before, the SI spoke was discarded for reconstruction. (D.) The k-space center magnitude for all four repetitions is shown for spokes acquired at the beginning of the sequence, before steady state was reached (these were discarded during acquisition). Odd and even repetitions exhibit opposite effects, with a small residual difference that corresponds to the zig-zag pattern in the phase seen in Figure 5. (E.) Importantly, when averaging magnitude differences (Rep 1 / Rep 3 and Rep 2 / Rep 4) across all interleaves in steady state, the continuous trajectory shows the smallest deviation for the first spokes at the start of each interleave, i.e., immediately after the “jump” in k-space. Since the first spoke (the SI spoke) is discarded during reconstruction, this explains why no substantial differences are observed between the two new trajectory designs in the final images.
